# Proteinous Components of Neutrophil Extracellular Traps Are Arrested by the Cell Wall Proteins of *Candida albicans* during Fungal Infection, and Can Be Used in the Host Invasion

**DOI:** 10.3390/cells10102736

**Published:** 2021-10-13

**Authors:** Justyna Karkowska-Kuleta, Magdalena Smolarz, Karolina Seweryn-Ozog, Dorota Satala, Marcin Zawrotniak, Ewelina Wronowska, Oliwia Bochenska, Andrzej Kozik, Angela H. Nobbs, Mariusz Gogol, Maria Rapala-Kozik

**Affiliations:** 1 Department of Comparative Biochemistry and Bioanalytics, Faculty of Biochemistry, Biophysics and Biotechnology, Jagiellonian University, 30-387 Krakow, Poland; justyna.karkowska@uj.edu.pl (J.K.-K.); magdalena.smolarz@doctoral.uj.edu.pl (M.S.); k.seweryn@doctoral.uj.edu.pl (K.S.-O.); dorota.satala@uj.edu.pl (D.S.); marcin.zawrotniak@uj.edu.pl (M.Z.); ewelina.wronowska@uj.edu.pl (E.W.); mariusz.a.gogol@gmail.com (M.G.); 2 Department of Analytical Biochemistry, Faculty of Biochemistry, Biophysics and Biotechnology, Jagiellonian University, 30-387 Krakow, Poland; olivia.bochenska@uj.edu.pl (O.B.); andrzej.kozik@uj.edu.pl (A.K.); 3 Bristol Dental School, University of Bristol, Bristol BS1 2LY, UK; angela.nobbs@bristol.ac.uk

**Keywords:** neutrophil extracellular traps (NETs), *Candida albicans*, fungal cell wall proteins, fungal adhesins, moonlighting proteins, granular proteins, elastase, myeloperoxidase, cathelicidin LL-37, histones

## Abstract

One of defense mechanisms of the human immune system to counteract infection by the opportunistic fungal pathogen *Candida albicans* is the recruitment of neutrophils to the site of invasion, and the subsequent production of neutrophil extracellular traps (NETs) that efficiently capture and kill the invader cells. In the current study, we demonstrate that within these structures composed of chromatin and proteins, the latter play a pivotal role in the entrapment of the fungal pathogen. The proteinous components of NETs, such as the granular enzymes elastase, myeloperoxidase and lactotransferrin, as well as histones and cathelicidin-derived peptide LL-37, are involved in contact with the surface of *C. albicans* cells. The fungal partners in these interactions are a typical adhesin of the agglutinin-like sequence protein family Als3, and several atypical surface-exposed proteins of cytoplasmic origin, including enolase, triosephosphate isomerase and phosphoglycerate mutase. Importantly, the adhesion of both the elastase itself and the mixture of proteins originating from NETs on the *C. albicans* cell surface considerably increased the pathogen potency of human epithelial cell destruction compared with fungal cells without human proteins attached. Such an implementation of adsorbed NET-derived proteins by invading *C. albicans* cells might alter the effectiveness of the fungal pathogen entrapment and affect the further host colonization.

## 1. Introduction

The fungal opportunistic pathogen *Candida albicans* resides as a commensal on the mucosal membranes and skin of most healthy individuals. However, under the reduced efficiency of the host immune system caused by functional defects of immune cells (neutropenia), chemotherapy, organ transplantation or the application of broad-spectrum antibiotics, *C. albicans* can cross cell or tissue barriers, leading to a variety of infections with different severity, ranging from relatively mild superficial mucosal infections to life-threatening fungemia and systemic diseases [1,2,3]. The efficiency in the colonization of different host niches is supported by the ability of *C. albicans* to switch its morphological form from unicellular yeast-like cells to filamentous forms (hyphae and pseudohyphae) associated with a wide range of virulence factors and mechanisms. They include the formation of biofilms, phenotypic switching, the expression of a number of adhesins on the cell surface, and the secretion of the aspartyl protease family with broad-spectrum activity [4,5,6].

Upon fungal infection, the human host recruits early neutrophil granulocytes as the most effective and efficient phagocytes to control and eliminate microbial invaders [7]. Contacting *C. albicans* cells, neutrophils engage the internal phagocytosis process and the extracellular release of granular content, or the formation of extracellular trap (NETs) containing decondensed chromatin. The latter, which is effective toward both the morphological fungus forms [8,9], is mostly activated by the NADPH oxidase signaling cascade [10,11], which delivers reactive oxide species (ROS) for the activation of azurosome. The released myeloperoxidase (MPO) and elastase (HNE) can translocate into the nucleus and degrade H1 histones with final chromatin expulsion [12].

The yeast cells to be internalized are processed by the phagocytosis mechanism, supported by the opsonization of the fungal surface with host proteins [13]. On the contrary to macrophage and murine neutrophils, in which *C. albicans* cells—after ingestion—can form hyphae within the phagocytes and—by outgrowth—can escape from them [14], the human neutrophils rather block *C. albicans* germination, probably owing to the higher activity of MPO and HNE [15]. On the other hand, the external process of NET release prevents the propagation of filamentous fungal forms by trapping them into excreted DNA fibers and exposing them to a milieu of highly concentrated antimicrobial compounds bound to the released DNA trap, including granular enzymes (MPO, HNE), antimicrobial proteins (lactoferrin, LF), azurocidin (AZU), and peptides like cathelicidin LL-37 [16,17,18,19]. This type of neutrophil response is activated due to the recognition of the main *C. albicans* virulence factors, especially glucans [20], mannans [21] and secreted proteases [9], as well as a quorum-sensing molecule farnesol [22], and extracellular nucleic acids present in fungal biofilms [23]. 

Extensive studies over the last few years focused on the mechanism of NET formation and NET’s ability to kill the fungal pathogen, but satisfactory data concerning the potent interaction of fungi captured in NET structures with the NET components have not yet been presented. However, such a problem could be of special interest during the search for a new, potent antifungal therapy, as the precise localization of *C. albicans* cells or their trapping may be the first step in the development and further application of drugs that can locally kill the fungal pathogen. In our current study, we identified possible interactions between proteins present on the surface of the fungal cells and proteinous NET components. We found that the main granular proteins and antibacterial peptide LL-37, as well as histones, interact with a typical glycosylphosphatidylinositol (GPI)-anchored adhesin of *C. albicans* from the agglutinin-like sequence family, Als3, and several proteins more loosely bound to the fungal cell wall (“moonlighting proteins” [24]), i.e., enolase (Eno1), phosphoglycerate mutase (Gpm1) and triosephosphate isomerase 1 (Tpi1). These interactions did not inactivate the NET antimicrobial enzymes like MPO and HNE, which modify the fungal cell surface, influencing their viability. On the other hand, the local modifications—such as citrullination or the proteolysis of NET components—at the place of their excretion could influence the antimicrobial potential of NETs, and could enable the escape and further dissemination of still-alive fungal cells. 

## 2. Materials and Methods

### 2.1. Yeast Strains and Culture Conditions

*C. albicans* cells of the ATCC 10231 strain from the American Type Culture Collection (Manassas, VA, USA) were cultured under different conditions, depending on the morphological form required for the experiments. The yeast-like form of *C. albicans* was grown in YPD medium (1% yeast extract, 2% soybean peptone and 2% glucose) (Sigma, St. Louis, MO, USA) for 16 h at 30 °C in an orbital shaking incubator MaxQ 6000 (Thermo Fisher Scientific, Waltham, MA, USA) (170 rpm), to the stationary phase. The hyphal form of *C. albicans* was obtained by the inoculation of the stationary phase cells (4 × 10^8^ of cells) into 20 mL of the RPMI 1640 medium (PAA Laboratories GmbH, Pasching, Austria), followed by further incubation with constant shaking (50 rpm) for 3 h at 37 °C for the mass spectrometry analysis, or 72 h (170 rpm) for the isolation of the surface proteins. Additionally, for the binding analyses, 1 × 10^6^ yeast-like cells were further grown in 150 µL RPMI 1640 medium for 3 h at 37 °C in the wells of CELLSTAR^®^ 96-well plates with flat micro-clear bottom black polystyrene wells (Greiner Bio-One, Kremsmünster, Austria). *Saccharomyces cerevisiae* strains expressing selected *C. albicans* adhesins were cultured without uracil in 20 mL of liquid complete synthetic medium (CSM) (Formedium, Norfolk, UK) with 0.67% yeast nitrogen base (Sigma) and 2% glucose (Sigma) at 30 °C for 24 h in an orbital shaking incubator (180 rpm), while the parent strain *S. cerevisiae* BY4742 was grown under the same conditions in the presence of 25 μg/mL uracil (Reanal, Budapest, Hungary) [25,26,27]. The list of the used strains is included in Supplementary Table S1.

### 2.2. Isolation of the Neutrophils

Fresh peripheral venous blood was collected from healthy volunteers via the Regional Blood Donation Center (Krakow, Poland), which complies with the requisite confidentiality assurances for human participants. Thus, the current work adheres to appropriate exclusions from human subjects’ approval. The blood sample was diluted in 5 mM EDTA solution (Sigma) and centrifuged at 300 g for 15 min to remove the plasma layer. The remaining suspension was diluted with Mg^2+^- and Ca^2+^-free phosphate-buffered saline (PBS) (Biowest, Nuaille, France) and overlaid on the lymphocyte separation medium (Biowest). After centrifugation at 300 g for 30 min, the pellet-containing neutrophils and red blood cells (RBCs) were subjected to 1% polyvinyl alcohol (Sigma) sedimentation to separate the RBCs from the neutrophils. Residual RBCs were lysed by Red Blood Lysis Buffer (Roche, Penzberg, Germany) and then washed with PBS. The number of cells was determined using a Bürker chamber. The neutrophils’ purity was assessed routinely by forward- and side-scatter flow cytometric analyses. This neutrophil isolation procedure yields a >95% pure population of the cells.

### 2.3. NET Production and the Degradation of Their Components

In order to verify the importance of NET components for *C. albicans* cell trapping, the following procedure was performed. Briefly, freshly isolated neutrophils were settled for 30 min in RPMI 1640 medium on 6-well plates (5 × 10^6^ cells per well) (Corning Inc., Corning, NY, USA) or 96-well microplates (2.5 or 5 × 10^4^ cells/well) (Cellvis, Sunnyvale, CA, USA) coated with 0.01% poly-L-lysine. Then, the cells were treated with 25 nM solution of phorbol 12-myristate 13-acetate (PMA) for 3 h at 37°C, 5% CO_2_, to induce NET production. NET formation was detected using SytoxGreen (Molecular Probes, Eugene, OR, USA) dye (excitation and emission wavelengths of 425 nm and 525 nm, respectively) at a final concentration of 1 μM, and fluorescence microscopy using an Olympus IX73 microscope (Olympus, Tokyo, Japan). Then, the NETs were treated for 1 h at 37 °C with (i) DNase (Thermo Fisher Scientific), 10 U/mL in PBS or (ii) proteinase K (Invitrogen, Carlsbad, CA, USA), 1 mg/mL in PBS. The activity of the protease was stopped by adding 5 µl 100 mM phenylmethylsulphonyl fluoride (PMSF) (Sigma). For further colocalization analysis, NETs which were not treated with proteinase K, characterized by the maximum ability to bind *Candida* cells, served as a control. After protein degradation, the plate was incubated for 1 h in the presence of 1% bovine serum albumin (BSA) (BioShop Canada Inc., Burlington, ON, Canada) to coat the unoccupied well surface, and was washed twice with PBS.

### 2.4. Colocalization Analysis

In total, 10^6^ *C. albicans* cells suspended in 100 µL RPMI 1640 without phenol red (Biowest) were added to the formed NETs (not treated/treated with proteinase K) and allowed to interact with their components for 1 h, at 37 °C and 5% CO_2_. *C. albicans* cells were stained with Calcofluor White dye (Sigma) at a final concentration of 1 μg/mL (with excitation and emission wavelengths of 350 nm and 460 nm, respectively). After incubation, the cells were washed twice with PBS. The colocalization of the yeast cells with NETs was performed using a fluorescence microscope IX73 (Olympus). For this purpose, every image was taken in two fluorescence channels (the green channel for NETs, and the blue channel for *Candida* cells). The analysis of the colocalization of signals from the green and blue channels was performed using Olympus cellSens Dimension 3.1 imaging software (Olympus), after deconvolution.

### 2.5. Identification of the Main NET Markers on the Surface of C. albicans with Fluorescence Microscopy

Neutrophils (5 × 10^4^ cells/well) were plated on a glass-bottom 96-well microplate (Cellvis) coated with 0.01% poly-L-lysine (Sigma) in 100 µL of RPMI 1640, and were allowed to settle for 30 min at 37 °C and 5% CO_2_. *C. albicans* cells (10^6^ cells/well) were added to PMA-treated (25 nM) neutrophils in 100 μL of RPMI 1640 medium, and were incubated for 3 h at 37 °C and 5% CO_2_. After neutrophil stimulation, DNase I (10 U/mL) was added to the formed NETs for further incubation for 1 h at 37 °C. Then, the digested DNA was removed by washing twice with PBS. The plate was incubated for 1 h in the presence of 1% BSA to prevent the non-specific binding of antibodies. Then, the cells were fixed with 4% paraformaldehyde for 10 min and washed with PBS. Next, 50 µL solutions of primary rabbit anti-MPO, anti-HNE, and mouse anti-H3 antibodies (Abcam, Cambridge, UK) diluted in PBS (1:100) were added to separate wells and incubated at 4 °C overnight. After incubation, the cells were washed three times with PBS and incubated with a 50 µL solution of secondary anti-mouse and anti-rabbit antibodies (Abcam) conjugated with Alexa Fluor 555 (1:300 dilution in PBS) for 1 h at 37 °C, and then washed three times with PBS. The *C. albicans* cells were stained with Calcofluor White dye at a final concentration of 1 μg/mL. The identification of the selected proteins (MPO, HNE, H3) on the surface of *C. albicans* was carried out using a fluorescence microscope (IX73, Olympus). Every image was taken in two fluorescence channels (red for neutrophils proteins, blue for *Candida* cells). 

### 2.6. Analysis of the NET Protein Interactions with the Surface of C. albicans Cells

For the determination of a possible interaction of the fungal cell surface with proteins included in the NET structure, two methods were used, depending on the morphological form of the *C. albicans* cells. The NET proteins MPO and HNE were supplied by Biocentrum (Kraków, Poland), LF was supplied by Sigma, and the histones were supplied by New England BioLabs (Ipswich, MA, USA). For the yeast-like form, flow cytometry analysis was used, and for the hyphae the microplate assay was applied. In the flow cytometry experiments, 1.3 × 10^6^ *C. albicans* cells were transferred into 1.5 mL Eppendorf tubes (pre-treated with 0.5% of BSA) and incubated for 1 h at 37 °C in the presence of the selected proteins, which were labeled previously with NHS-fluorescein (Thermo Fisher Scientific, Waltham, MA, USA) according to supplier protocol. Then, the cells were washed three times with PBS and analyzed on a BD Fortessa flow cytometer (BD Biosciences, Franklin Lakes, NJ, USA), and using FACS Diva (BD Biosciences) and FlowJo 8.7 software (BD Biosciences). The results were presented as a mean fluorescence intensity per cell.

In the microplate assay, the binding of fluorescein-labeled NET proteins was performed on *C. albicans* hyphal forms (1 × 10^6^ cells) grown in RPMI 1640 medium for 3 h at 37 °C in the wells of CELLSTAR^®^ 96-well plates (Greiner Bio-One). The unoccupied well surface was blocked with 3% BSA in PBS. After the wells’ washing, the solution of the selected, labeled proteins in PBS was added, gently mixed, and incubated for 1.5 h at 37 °C. The amount of bound protein was determined by the measurement of the fluorescence intensity (excitation and emission wavelengths of 488 nm and 525 nm, respectively) with a multi-mode microplate reader Synergy H1 (BioTek, Winooski, VT, USA). For both types of experimental data, the protein binding analysis was performed using GraphPad Prism version 8.0 (GraphPad Software, San Diego, CA, USA) and a saturation one-site binding model.

### 2.7. Extraction and Purification of C. albicans Cell Wall Proteins

The extraction of the cell wall-associated proteins (CWP) from *C. albicans* hyphae was performed according to the previously described method [28,29]. Briefly, the protein extracts were obtained by treating the cells with β-1,3-glucanase (Sigma) and 2-mercaptoethanol (Sigma) in the presence of protease inhibitor cocktail (Roche, Basel, Switzerland). In order to confirm the cell viability during the isolation procedure, Trypan Blue (Sigma) staining was used, which showed that more than 98% of the cells remained viable. The CWP-containing extracts, collected after the cell removal by centrifugation, were dialyzed against PBS (48 h at 4 °C) and used for (i) the identification of particular fungal proteins interacting with NET proteinous components by chemical cross-linking, and (ii) for the purification of selected fungal surface proteins―Als3 and Tpi1. The purification process involved (i) high-performance ion-exchange chromatography on a MonoQ column (GE Healthcare/Pharmacia, Uppsala, Sweden) and high-performance gel filtration on a Superdex-200 column (Amersham Bioscience, Little Chalfont, UK), as described in detail in previous work [28]. The purification of the enolase (Eno1) was performed according to an already published method [30], in which the starting material was a cytosolic fraction of *C. albicans* cells. The purification involved two steps of ion-exchange chromatography with the application of MonoQ and MonoS columns (GE Healthcare/Pharmacia, Uppsala, Sweden). Phosphoglycerate mutase 1 (Gpm1) was heterologously expressed and overproduced in *Escherichia coli* as His-tagged proteins, pu-rified to homogeneity using affinity and ion chromatography steps [28]. The protein concentration was measured by the method of Bradford [31]. The purity of all of the isolated proteins was confirmed by SDS-PAGE in the Laemmli system [32], and by liquid chromatography-coupled tandem mass spectrometry (LC-MS/MS) using a Dionex UltiMate 3000 UHPLC system (Dionex, Carlsbad, CA, USA) coupled with an HCT Ultra ETD II mass spectrometer (Bruker, Bremen, Germany), as described in detail in [28].

### 2.8. Binding of the NET Proteins to Selected C. albicans Adhesins Expressed at the Surface of S. cerevisiae Cells

For the analysis of the binding of particular NET proteins to selected *C. albicans* adhesins expressed at the cell surface of *S. cerevisiae* cells, flow cytometry analysis was applied similarly to the method described previously in [33]. Briefly, 5 × 10^6^ yeast cells were transferred into 1.5 mL Eppendorf tubes, and incubated for 1.5 h at 37 °C in the presence of 50 µL solutions of selected NET proteins—LF labeled with NHS-fluorescein, HNE, MPO, and histone 3 at a concentration of 200 nM. In the case of the HNE solution, it was preincubated with 10 µM elastase inhibitor (*N*-(Methoxysuccinyl)-Ala-Ala-Pro-Val- chloromethyl ketone, Sigma) for 15 min at 37 °C. Then, the 50 µLsolutions of primary rabbit anti-MPO, anti-HNE, and mouse anti-H3 antibodies (Abcam) diluted in PBS (1:100) were added to the cells, and the cells were incubated at 4 °C overnight. After the incubation, the cells were washed three times with PBS and incubated with secondary anti-mouse and anti-rabbit antibodies (Abcam) conjugated with Alexa Fluor 488 diluted 1:300 in PBS, for 1 h at 37 °C, and then washed three times with PBS. Next, the cells were incubated with 100 µL 4% paraformaldehyde for 15 min, washed three times with PBS, transferred to glass tubes and placed at 4 °C. On the following day, the cells were analyzed on a BD Fortessa flow cytometer (BD Biosciences, Franklin Lakes, NJ, USA), and using FACS Diva (BD Biosciences) and FlowJo 8.7 software (BD Biosciences). The results were presented as the mean fluorescence intensity per cell, after considering the relative differences in the surface exposition of particular adhesins, assessed using a 1 μg/mL solution of mouse anti-HA antibody (ZYMED Laboratories, South San Francisco, CA, USA) and anti-mouse Alexa Fluor 488-conjugated secondary antibodies (Abcam), as described above.

### 2.9. Quantitative Characteristics of Fungal CWP Binding to NET-Composing Proteins Using Surface Plasmon Resonance (SPR) Measurement 

The SPR measurements were performed using a BIACORE 3000 system (GE Healthcare, Uppsala, Sweden) in a running buffer (RB) containing 10 mM HEPES, 150 mM NaCl and 0.005% (*w*/*v*) surfactant P20, at pH 7.4. The purified CWP were immobilized on the surface of a CM5 sensor chip (GE Healthcare) according to the standard amine-coupling procedure, using 1-ethyl-3-(3-dimethylaminopropyl)carbodiimide (EDC), *N*-hydroxysuccinimide (NHS) and ethanolamine (Amine Coupling Kit, GE Healthcare). The reference cells, after activation, were directly blocked with ethanolamine. The immobilization of Tpi1 and Eno1 reached the level of 400 resonance units (RU), Gpm1 reached 300 RU, and the immobilization of Als3 reached 800 RU. In order to test the binding of NET-composing proteins, the protein solutions, at concentrations within a range of 2–2500 nM, were injected over the reference and sample cells, at a flow rate of 30 μL/min, with 2 min contact time for the association and 2 min for the dissociation. The regeneration of the sensor surface was performed with the application of two injection pulses (30 s, 90 μL each) of 1 M NaCl solution. Only the interaction between Als3 and MPO needed the regeneration with 0.1% SDS for 10 s. Then, the chip surface was washed with RB until a stable baseline signal was obtained. The measurements were repeated three times for each sample. The collected data were analyzed using BIAevaluation software version 4.1 (GE Healthcare). The resulting dataset were analyzed by the simultaneous fitting of the association rate constant (ka), the dissociation rate constant (kd) and the equilibrium dissociation constant (K_d_) to the 1:1 Langmuir binding model with a drifting baseline. 

### 2.10. Analysis of the Possible Modification (Citrullination or Proteolytic Degradation) of NET-Composing Proteins at the Place of Interaction with C. albicans Cells, and Its Influence on the Analyzed Interactions

In order to obtain the deiminated form of the histones, the proteins were incubated in the presence of 0.5 U protein arginine deiminase 4 (Pad4) (Sigma) in 100 mM Tris-HCl, at pH 7.4, supplemented with 10 mM CaCl_2_ and 2 mM DTT for 4 h at 37 °C. Then, the reaction was stopped by the addition of 20 mM EDTA solution. The citrullination of the histones was confirmed using mass spectrometry analysis using an HCT Ultra ETD II mass spectrometer (Bruker), as described previously [34]. For the analysis of the possible influence of the citrullination of peptide LL-37 (Sigma) on the interaction with fungal proteins, the modified peptides from the Biotechnology Core Facility Branch (Atlanta, GA, USA) were used, with the modification of the selected arginine residues, as follows: LL-37(7)Cit, LL-37(7,29)Cit, LL-37(7,29,34)Cit, LL-37(7,19,23,29,34)Cit.

The deiminated histones or LL-37 and their unmodified references were treated with 6 nM *C. albicans* recombinant secreted aspartyl proteinases (Sap), Sap3 and Sap9, expressed and purified—as described previously in detail in [35]—in 50 mM citrate buffer (pH 5) at 37 °C for 1 h at a 1:1000 enzyme:substrate molar ratio. The enzymatic reaction was stopped with the addition of pepstatin A (Amresco, Solon, OH, USA) at the final concentration of 10 µM.

The products of the degradation of both the proteins and peptides were separated under reducing conditions by SDS-PAGE in the Schägger von Jagov system [36]. The protein bands representing the degradation products were visualized by the silver staining method using fixation in 10% glutaraldehyde for 30 min [37].

### 2.11. The Influence of the Binding of NET Proteins to the Fungal Cell Surface on the Interactions with Epithelium 

Adenocarcinomic human alveolar basal epithelial cells (A549) were grown in F12-K medium (Corning) supplemented with 10% fetal bovine serum (Gibco, Billings, MT, USA) at 37 °C and 5% CO_2_ in a humidified chamber. A glass-bottomed 96-well plate (Cellvis) was coated with 0.05 mg/mL collagen from the human placenta (Sigma), and the incubation continued for 2 h. After this time, 6.5 × 10^5^ cells per plate were incubated overnight at 37 °C and 5% CO_2._ Then, the epithelium monolayer was co-incubated with *C. albicans* cells bound to NET proteins or HNE. For this purpose, HNE at a final concentration of 14 µg/mL and NET proteins at a concentration of 212 µg/mL were added to 10^6^ *C. albicans* cells, and were incubated for 1 h at 37 °C. The cells were then washed 3 times with PBS and added to the epithelium monolayer (10^4^ cells/well). Co-incubation was carried out for 24 h. The viability of the A549 cells was assessed with MitoTracker™ Green FM dye (at a final concentration of 1 µM). The cells were imaged using an IX73 microscope.

### 2.12. The Influence of HNE Binding to the Fungal Cell Surface on C. albicans’ Viability

In order to evaluate the effect of HNE binding on *C. albicans* viability, HNE (a final concentration of 14 µg/mL) was added to the cells grown in the hyphal form (10^6^ cells/mL) on a 96-well plate and incubated in HNE solution for 1 h. The cells were then washed 3 times with PBS, and the incubation was carried out for an additional 1 h or 24 h only with bound HNE. The cell viability was assessed by the XTT (sodium 3′-[1-(phenylaminocarbonyl)-3,4-tetrazolium]-bis (4-methoxy6-nitro) benzene sulfonic acid hydrate) test. For this purpose, the cells were washed 2 times with PBS and then 50 µL of a mixture containing XTT (a final concentration of 1 mg/mL), and PMS (*N*-methyl dibenzopyrazine methyl sulfate, at a final concentration of 5 µg/mL) was added. After 3 h of incubation at 37 °C, the absorbance was measured at 450 nm using a Synergy H1 microplate reader. Additionally, yeast viability was tested using a CaspACE™ FITC-VAD-FMK In Situ Marker (Promega, Madison, WI, USA) according to the manufacturer’s protocol. Briefly, a dye at the final concentration of 10 µM in PBS was added to the cells and incubated for 30 min. Subsequently, the cells were washed 4 times with PBS and imaged using an IX73 fluorescence microscope in the FITC channel. 

## 3. Results

### 3.1. Neutrophil Proteins Located within the NET Structures Are Involved in the Capturing of C. albicans Cells

**Figure 1:**
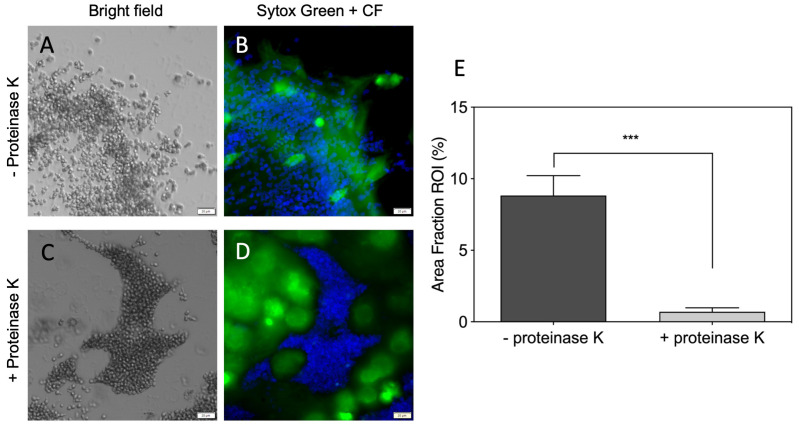
A significance of NET-related proteins for the interaction with *C. albicans* cells. In total, 2.5 × 10^5^ neutrophils were stimulated for 3 h with 25 nM PMA. The extracellular DNA was stained with Sytox Green. The neutrophil proteins were eliminated from the NETs with proteinase K treatment (**C**,**D**). NETs which were not treated with proteinase K served as a control (**A**,**B**). In total, 10^6^ *C. albicans* cells labeled with Calcofluor White (CF) were added to the released NETs (treated/not treated with proteinase K) and incubated for 1 h. A colocalization of yeast cells and NETs was assessed qualitatively using Olympus IX73 fluorescence microscopy (**A**–**D**, cropped) and quantitatively using the Olympus cellSens imaging software. The colocalization value is presented as the percentage of the image area where the blue fluorescence channel collocates with the green one (**E**). A representative result of three independent experiments is presented. In order to assess the statistical significance between groups, an unpaired *t*-test was performed. The results were considered statistically significant at a *p*-value of <0.05 (*** *p* ≤ 0.0002).

**Figure 2:**
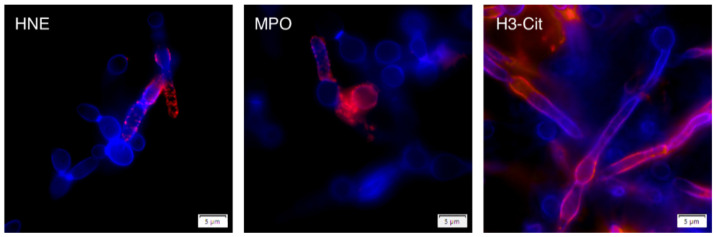
The localization of NET proteins on the hyphal forms of *C. albicans* cells after the DNA scaffold removal. Neutrophils (5 × 10^4^ cells) were incubated with *C. albicans* (10^6^ cells) for 3 h at 37 °C. After stimulation, the released NETs were degraded by DNase I (5 U/100 µL). The yeast-bound neutrophil proteins were identified using the rabbit primary antibodies anti-HNE, anti-MPO, and mouse anti-histone H3 or anti-citrullinated histone H3 (1:100), and then secondary Alexa Fluor 555-labeled antibodies (1:300). The *C. albicans* cell wall was stained with Calcofluor White dye (blue). A representative result of three experiments is presented. The presence of HNE, MPO and citrullinated histone H3 on the surface of *C. albicans* was visualized with a Olympus IX73 fluorescence microscope (60× magnification).

The possible killing of *C. albicans* cells by neutrophil-forming NETs was detected upon the contact of the alarmed host cells with the yeast-like and hyphae forms of the *C. albicans* cells [38]. Some data documented that the entrapped microorganisms are still alive, but NETs prevent their further dissemination [39]. As it is generally believed that DNA fibers are mainly responsible for microbe trapping and its entangling, in the present work we analyzed the role of proteinous components of NETs during the recognition and entrapment of *C. albicans* cells. In order to decide which of the components of freshly created NETs upon PMA treatment is important for the entrapment of the interaction with fungal cells, we applied treatments of NETs with protease K and DNase to remove the proteins or DNA, respectively, from the NET structures (Figure 1 and Figure 2). Such prepared NETs, as well as the untreated NETs serving as a reference, were further analyzed for *C. albicans* cell adhesion. The proteinase K treatment of the NETs eliminated the protein components from these fibers, as was proven by the testing of the remaining DNA structures with the antibodies recognizing the main protein components of NETs: histones, MPO and HNE. The remaining DNA structures, deprived of proteins, were blocked with BSA and tested for *C. albicans* cell binding (Figure 1A–D). The fungal cells, mainly in their yeast form, almost did not colocalize with the remaining DNA fibers (Figure 1E). Instead, the fungal cells were partially located on the microplate surface, omitting the DNA-presented scaffold. These results pointed rather at the proteinous components of the NETs as the main target for their interactions with fungal cells. 

In order to verify such a conclusion, we treated NETs, formed during 3 h of contact with *C. albicans* cells, with DNase to eliminate DNA fibers, and probed the surface of the fungal cells, remaining on the microplate, with the antibodies to identify the selected proteinous NET components: MPO, HNE, histone H3 and its citrullinated form (Figure 2). During the contact of the neutrophils and *C. albicans* cells, the fungal cells developed the hyphae, which are more resistant to neutrophil actions. On their surface, the antibody treatment localized MPO and HNE but not the selected histone H3; instead, the modified form of the latter was detected. These results supported the hypothesis of the important role of the proteinous components of NETs for fungal cell entrapment by these neutrophil structures.

However, in the above-demonstrated study, we perform the identification of only the selected NET-composing proteins. In order to extend the range of the NET proteins studied, in further experiments we used the whole NET protein extract received after the DNase treatment of the NETs and the neutrophil cell debris removal. In order to identify their interaction with the *C. albicans* cell surface, both the blastospores and filamentous forms were incubated with NET-composing proteins for 1 h at 37 °C in PBS buffer. The bound proteins were analyzed using the mass spectrometry method, after cell surface shaving with trypsin [40]. The identified neutrophil proteins involved in the interactions with the fungal cell surface were represented mainly by granular proteins: MPO, HNE, cathepsin G, proteinase-3, lactotransferrin, and cathelicidin-derived peptide LL-37, and additionally histones (Supplementary Table S2). Furthermore, some cytosolic proteins were detected, especially protein S-100 (calprotectin), which was previously identified as possessing antifungal properties [38], and cytoskeleton proteins, such as actin, vimentin and myosin. 

**Figure 3:**
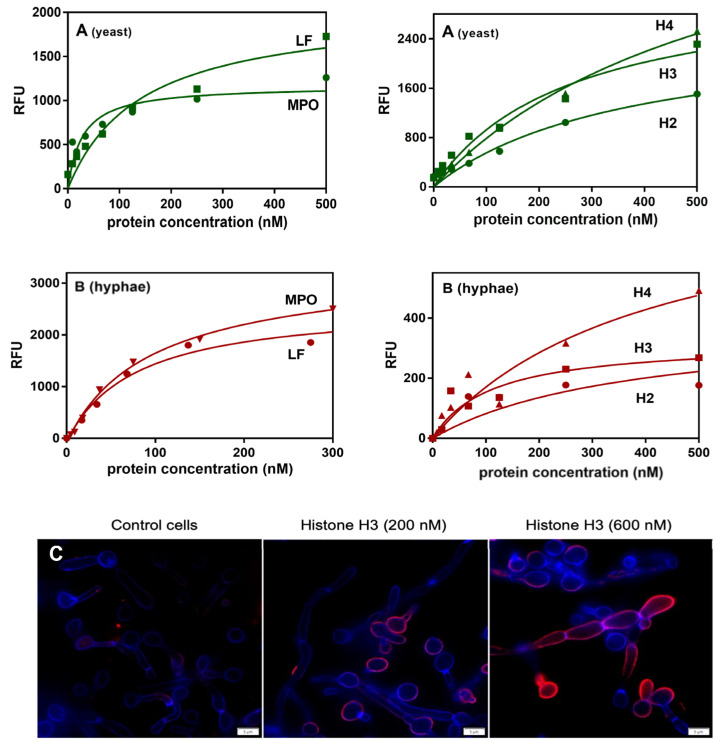
Interactions of selected NET proteins with the yeast and hyphal forms of *C. albicans* cells. The interactions of commercially available NET-composing proteins were tested using flow cytometry analysis (**A**) for the yeast-like form, and the microplate ligand-binding assay (**B**,**C**) for the hyphae. In the flow cytometry experiments, 1.3 × 10^6^ *C. albicans* cells were incubated in the presence of selected proteins, previously labeled with NHS-fluorescein. The results were presented as a mean fluorescence intensity per cell. In the microplate assay, the binding of fluorescein-labeled NET proteins was tested towards *C. albicans* hyphal forms (1 × 10^6^ cells) grown in RPMI 1640 medium for 3 h at 37 °C. After washing and blocking the unbound surfaces, the cells were incubated with labeled NET proteins. The amount of bound proteins was determined by measurements of the fluorescence intensity (excitation and emission wavelengths of 488 nm and 525 nm, respectively) with a multi-mode microplate reader. For all of the experimental data, the protein binding analysis was performed using GraphPad Prism software and a saturation one-site binding model. The representative result of three experiments performed in duplicates is presented. The microscopic analysis (Olympus IX73 microscope) of histone H3 binding to the surface of *C. albicans* cells was performed, after the cell fixation, using mouse primary anti-H3 antibodies and Alexa Fluor 555-conjugated secondary antibodies. The fungal cell wall was labeled with Calcofluor White dye.

Next, the binding of the purified NET proteinous components to the yeast and hyphal forms of *C. albicans* cells was verified using flow cytometry analysis or an ELISA-like microplate ligand-binding assay, respectively (Figure 3). For both morphological forms of *C. albicans,* the binding of selected granular components of the NETs was observed, with the strongest effect for MPO in the binding to the yeast form, and lactotransferrin and MPO for binding to hyphae, as was concluded from the saturation pots that allowed us to estimate the apparent binding constants as the neutrophil protein concentration necessary for the half-saturation of cell binding surfaces. We also examined a possible binding of histones to the fungal cells in both morphological forms, and also found these proteins, especially histone 3 contacting hyphae, as a potential target for such an interaction with *C. albicans* cells. On the other hand, the comparison of the protein-binding properties for both types of *C. albicans* cells suggested that the natures of the binders for histones on both cell surfaces were nearly the same. On the contrary, the binding properties for granular proteins differed between the cell types, suggesting the involvement of different classes of binders or receptors on the surface of both of the morphological forms of fungal cells. As presented in the panel C of Figure 3, the binding of uncitrullinated histone H3 was also possible, and no differences were detected in the shapes of the binding plots, illustrating the interactions with both forms of this protein (data not presented). 

### 3.2. Identification of the Fungal Surface Proteins Involved in the Interactions with Proteinous NET Components

Chemical crosslinking was applied to search for the fungal surface proteins that interact with the host’s proteinous partners located with NET structures. The integrity of the fungal cells during the surface protein isolation was verified in order to exclude contamination with cytoplasmic proteins, as was described by Seweryn et al. [28]. Particular NET proteins, chemically bound with the crosslinker sulfo-SBED, were incubated with a solution of candidal cell-wall-associated proteins isolated from the surface of the hyphal form of *C. albicans* cells. After 2 h of their interaction, the mixtures were exposed to UV light to perform the covalent linkage with the second interacting protein using the photoactive part of the crosslinker molecule. After the separation of the formed complexes on the magnetic beads, the bound fungal proteins were recovered using a reduction of disulfide bridge, located inside of the crosslinker arm. The obtained fungal proteins were identified using mass spectrometry (Supplementary Table S3). Among them, mainly the “atypical” candidal surface proteins were identified, with Eno1 (enolase) and Tpi1 (triosephosphate isomerase) being found to interact with all of the of the tested NET proteins; Gpm1 (phosphoglycerate mutase) being found to shun two enzymes, MPO and HNE; and Eft2 (translation elongation factor 2) being found to bind with the H3 and H4 histones, MPO and LF. No typical adhesins were identified with this method, probably because they are large and highly glycosylated proteins, which could not only make it difficult to interact with the cross-linker used but also hinder their subsequent release and identification.

**Figure 4:**
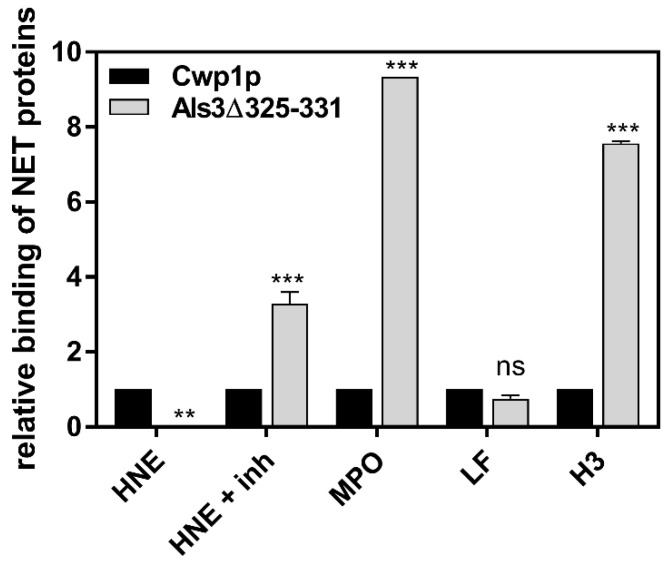
Binding of selected NET-composing proteins to *S. cerevisiae* cells bearing surface-expressed candidal Als3 adhesin. *S. cerevisiae* cells (5 × 10^6^ cells) overexpressing the *C. albicans* adhesins—Als3 and Als3Δ 325-331, and a control protein—Cwp1, were incubated in PBS buffer, at pH 7.4, for 1.5 h at 37 °C, with 50 μL 200 nM solutions of selected NET proteins: LF, MPO, H3, and HNE treated (+inh) with inhibitor for 15 min at 37 °C or uninhibited. The protein binding was determined using the recognizing primary antibodies and secondary antibodies conjugated with Alexa Fluor 488, except for LF pre-labeled with NHS-fluorescein. The amount of bound proteins was determined using fluorescence detection with the BD Fortessa flow cytometer and FlowJo software. The exposition of all of the fungal proteins was determined by the identification of HA-conjugate, detected with mouse anti-HA antibody. The results present the fold change of the signal compared with unspecific binding to Cwp1-exposing cells. The experiment was performed in triplicate, and cumulative results representing the means ± SEM were generated with GraphPad Prism software version 8.0 (GraphPad Software, La Jolla California USA) with a one-way ANOVA test with Dunnett’s multiple comparison test. The statistical significance levels were marked with ** for *p* < 0.01, *** for *p* < 0.001 and ‘ns’ for ‘not significant’.

While the fungal proteins identified by cross-linking as interacting with individual NET proteins were extracted from the surface of fungal cells, such that had not been in contact with the neutrophils, we decided to verify whether these proteins are also localized on the fungal surface under such conditions of the co-incubation of *C. albicans* filamentous forms with neutrophils, responding with NET formation. After the 4-h contact of fungi with neutrophils, the latter were removed, their remnants washed away and the fungal proteins were obtained from prepared cell walls using sodium dodecyl sulfate (SDS) and β-mercaptoethanol treatment (Supplementary Table S4). The removal of cytoplasmic contamination was achieved by using a series of cell wall washing steps, as described by Pitarch et al. [41]. *C. albicans* cells that did not contact with neutrophils served as a control. The comparison of the surface proteome of *C. albicans* hyphae with their form on contact with netting neutrophils revealed the presence of Eno1, Gpm1, Eft2, and Tpi1 in both conditions, which empowered us to further investigate the host–pathogen interactions with these particular proteins. Interestingly, of the several surface proteins identified under the experimental conditions used, a large proportion were atypical cell wall proteins or enzymes involved in cell wall remodeling, and the only surface-located protein being a typical adhesin identified at the surface of fungal cells after contact with neutrophils was Als3, which represents *C. albicans* adhesin with a broad spectrum of bound ligands belonging to different groups of human proteins [42]. Therefore, we also included this protein in our further studies of interactions with individual NET proteins. The data supporting such an approach were obtained from experiments in which *S. cerevisiae* surrogate cells exposing on their surface the *C. albicans* Als3 molecules as fusion proteins were used [26,27] for the testing of selected NET protein binding (Figure 4). The level of Als3 exposition was determined using specific antibodies recognizing a hemagglutinin (HA) tag localized near the C-terminus of fused protein. In order to simplify the analysis using the FACS method, the *S. cerevisiae* cells with expressed Als3 protein deprived of its amyloid sequence were employed (Als3Δ325–331), as this variant presented the same NET protein binding properties as the full-length Als3 protein (representative data for LF are presented in Supplementary Figure S1). The results were normalized to the values obtained for *S. cerevisiae* with the overexpression of Cwp1 protein, which did not show any significant specific binding of selected NET proteinous components. The final results qualified Als3 as a factor interacting mainly with MPO and histone H3. The binding of HNE was sensitive to its proteolytic properties, and the binding of LF remained on a level similar to the non-specific interactions.

### 3.3. Kinetic Characteristics of Protein–Protein Interactions between Selected NET-Composing Proteins and Candidal Adhesin Als3 and the Yeast Moonlighting Proteins

**Figure 5:**
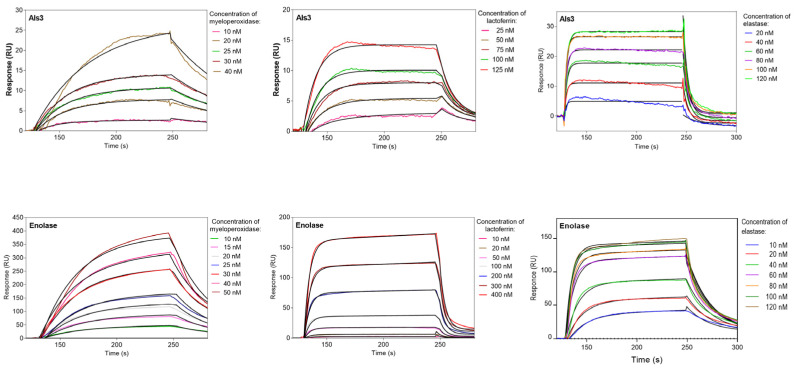
Binding of selected NET-composing proteins to immobilized fungal cell surface proteins―Als3 and enolase. The kinetic analysis of the binding properties of selected fungal proteins was performed using a BIACORE 3000 system with the detection of surface plasmon resonance changes (RU) during the NET-protein binding to the immobilized Als3 and enolase on the surface of CM5 chips. For the protein immobilization, the standard amine-coupling procedure was used. The immobilization level was 800 RU and 400 RU for Als3 and enolase, respectively. The selected host protein solutions were injected over the chip surface with a flow rate of 30 µL/min, for 2 min for association and 2 min for dissociation processes. For the chip surface regeneration, a pulse of 1 M NaCl (30 s) or 0.1% of SDS (10 s) was used, according to the manufacturer-recommended procedure. The data from three independent tests were analyzed by BIAevaluation software version 4.1 (GE Healthcare). For each dataset, the simultaneous fitting of constants for association and dissociation rates was used with the 1:1 Langmuir binding model.

In order to ascertain the interaction characteristics of the identified *C. albicans* cell wall proteins contacting NET-forming proteinous partners, the dissociation constants were determined for interacting protein–protein pairs using the microplate ligand-binding assay, or SPR measurements in a BIACORE 3000 system. The fungal proteins used in these experiments were purified based on the fractions identified in ion chromatography on the MonoQ column, with the further application of size exclusion chromatography for the complete purification of Als3 and Tpi1. Eno1 was isolated from the cytosolic proteins fraction, as no differences so far were identified between these cytosolic and surface-located proteins [43], as confirmed by mass spectrometry analysis. Gpm1 and Eft2 were overexpressed in *E. coli*, but this process was successful only for Gpm1, which was further purified by affinity and ion-exchange chromatographic steps, as described above. For the analysis of mutual interactions, the *C. albicans* CWP were immobilized on the surface of the microplate for the ELISA-like assay, or the surface of CM5 microchips for the SPR measurements. The representative sensograms for interactions between a typical adhesin Als3 and enolase―one of the best-known moonlighting proteins in many organisms―are presented in Figure 5 for the tested granular components. All of the analyses were carried out in a wide range of analyte concentrations, and the significant changes in responses for interacting pairs are presented. As a negative control for the host protein binding to immobilized Als3 we used prekallikrein, which is known not to interact with Als3, as was demonstrated by Seweryn et al. [28]. However, we did not have at hand such type of a control for enolase.

A similar analysis for histones was impossible to perform due to their high level of nonspecific binding to the chip surface. Therefore, in this case, the microplate approach was used for all of the *C. albicans* proteins with exception of Tpi1, which was isolated in amounts which were too small to perform these types of experiments, in which the binding of fluorescent staining proteins was analyzed. All of the determined K_d_ parameters are summarized in Table 1. However, it should be kept in mind that K_d_ values determined using different methods should not be directly compared.

The comparison of them confirmed the identified earlier main involvement of the fungal adhesin Als3 in the MPO binding. All of the analyzed neutrophil proteins were able to interact with Als3 in the following order of decreasing binding strength: MPO > H3 > HNE >LF > LL-37 >> H4, H2A. No significant influence on the enzymatic activity of MPO or HNE, even at a 10-fold molar excess of Als3 or moonlighting proteins, was detected (Supplementary Figure S3). Moreover, Als3 presented the strongest binding to histone H3 among all of the fungal proteins. 

The best-characterized *C. albicans* moonlighting protein, Eno1, was also involved in the interaction with NET proteins, but with a different order of preferences: LL-37 > HNE > LF, MPO >> H4 > H2A > H3. Its binding properties increased up to 10-fold in the presence of magnesium ions for all of the analyzed granular proteins (Supplementary Figure S2). It is worth noting that our data, for the first time, showed that atypical surface proteins like Eno1 can interact with LL-37 and HNE. On the analysis of the interactions with histones, Eno1 was found to serve also as a receptor, mainly for H4, but these binding properties in terms of the dissociation constant were about 20-times weaker compared with the LL-37 complex with Eno1 (Table 1). The next most potent fungal binder of NET proteins was Gpm1, interacting mainly with LF (LF >> H3 > MPO > LL-37 >> H4). Its binding properties towards histones were also interesting, as Gpm1 was a strong binder for H3 and much weaker for H4, while it was indifferent to H2. Another moonlighting protein tested―Tpi1―interacted most strongly with MPO and LL-37 among the analyzed granular proteins. However, its interaction with histones remains unconfirmed.

In summary, the identified *C. albicans* surface proteins can serve as the target for binding by human proteins that decorate NET structures. As these proteins are destructive not only towards the fungal cells but also can act against host cells, in the further experiments we considered their possible action during contact with both cell types. 

### 3.4. NET Proteins Bound to the Surface of Fungal Cells Do Not Eliminate Them but Rather Induce the Apoptosis of the Host Cells Surrounding the Infection Locus 

Granular proteins present in the released NETs are regarded as killing agents against the yeast cells trapped in the neutrophil network, but their efficacy in this form is only partial [38]. At the same time, yeasts are capable of producing DNases, which, by eliminating the main building blocks of NETs, enable the release of yeast cells from this trap. However, a question arises as to whether yeasts are only victims of the netosis process, or whether they can additionally benefit from it, especially since, as we have shown, the binding of the proteinous components of NETs to the fungal cell surface occurred. In order to address this question, we initially examined whether bound NET proteins, mainly HNE at its initial concentration of 500 nM in the fungal cell-contacting solution, can significantly influence cell viability. We chose elastase as a representative protein because of the simplest action mechanism, which remains undisturbed upon binding to yeast cells. Moreover, at the candidal infection site, the possible inhibition of HNE activity by its natural inhibitor, antitrypsin, may be abolished by the candidal proteases (Saps) [44].

**Figure 6:**
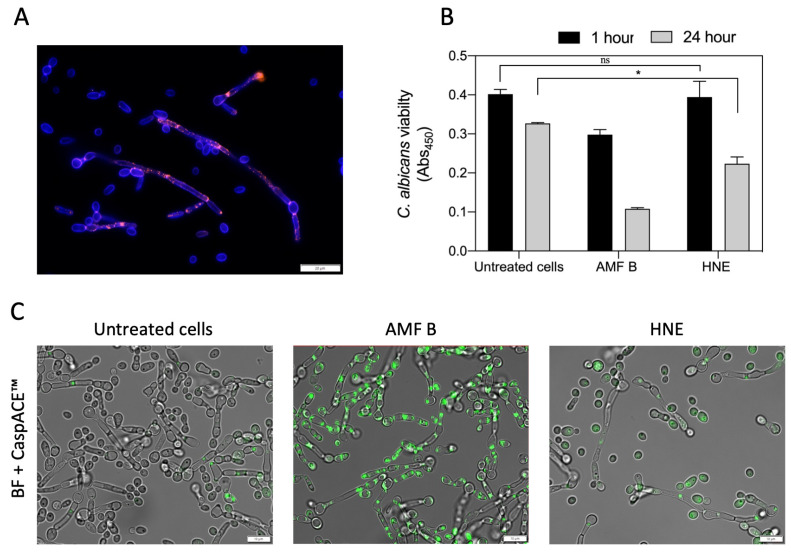
The effect of human neutrophil elastase binding on the viability of *C. albicans*. (**A**) *C. albicans* (10^5^ cells/mL) cells were grown in a 96-well plate in RPMI medium for 6 hours. HNE was added to the cells at a final concentration of 14 µg/mL. In order to visualize the bound HNE, the cells were washed with PBS and fixed with 4% PFA. The HNE presence on the fungal cells was identified using mouse anti-HNE antibodies (1:100) and secondary anti-mouse antibodies (1:300) conjugated with Alexa Fluor 555 dye. The *C. albicans* cell wall was stained with Calcofluor White dye. (**B**) An XTT test was performed to determine the viability of *C. albicans* after 1 or 24 h of incubation with HNE. Cells treated with amphotericin B (AMF B; 2.5 µg/mL) served as a positive control. A representative result of three independent experiments is presented. In order to assess the statistical significance between groups (*n* = 2), an unpaired *t*-test was performed. The results were considered statistically significant at a *p*-value of < 0.05 (* *p* ≤ 0.0332), or not statistically significant (ns) for *p* > 0.1234. (**C**) In addition, the viability of *C. albicans* was determined using the CaspACE ™ FITC-VAD-FMK In Situ Marker. The imaging was performed with an Olympus IX73 microscope in the FITC channel.

As was shown in Figure 6, purified HNE was bound mainly to the surface of *C. albicans* hyphae (Figure 6A), similarly to its detection after its contact with whole NET proteins (Figure 2). After removing the unbound host protein, the analysis of the further influence of its bound form on fungal cell viability showed that 75% of the cells remained alive even after 24 hours of contact, as was determined using the XTT assay (Figure 6B) and by the detection of metacaspase activity, in which amphotericin B was used as a positive control (Figure 6C). 

**Figure 7:**
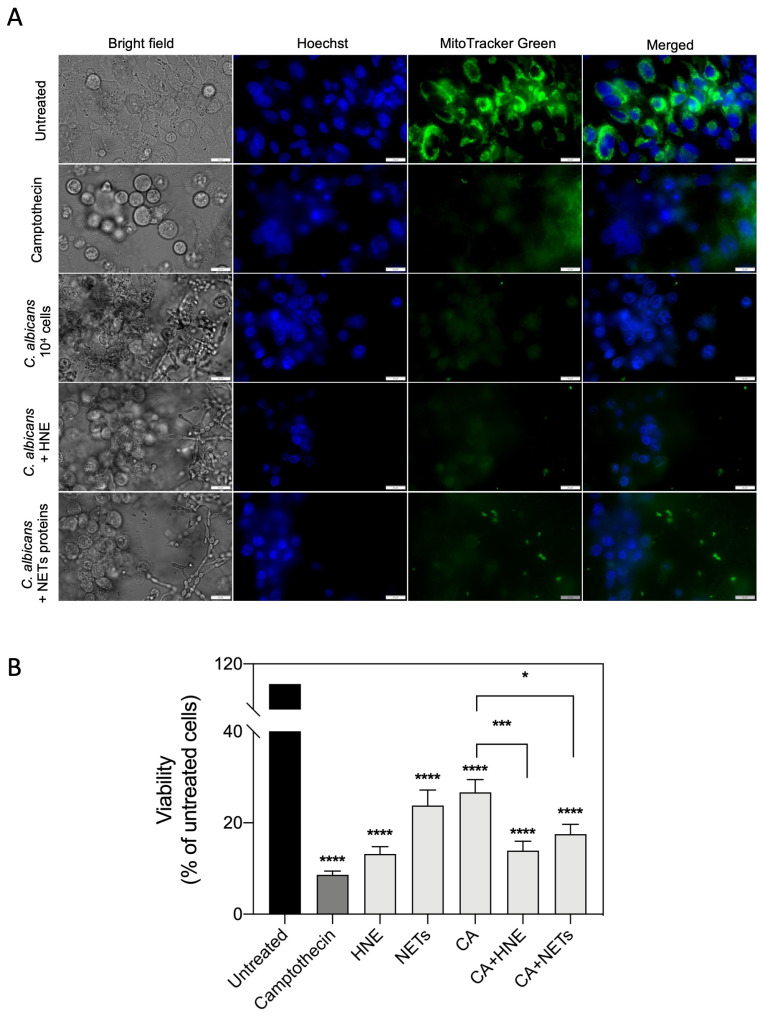
The influence of the binding of NET proteins to the *C. albicans* cell surface on the interactions with epithelial cells. *C. albicans* (10^6^ cells/mL) cells were incubated with NET proteins (212 µg/mL) and HNE (14 µg/mL) for 1 h at 37 °C, washed three times with PBS, and then added to a monolayer of A549 cells (10^4^ cells/well). Unstimulated cells served as a negative control, and cells treated with camptothecin (CA, 7 µM) were used as a positive control. The monolayers were incubated with all of the stimulants for 24 h at 37 °C and 5% CO_2_. Then, the cells were washed, and the viability was assayed using MitoTracker™ Green FM dye. The cell nuclei were stained using Hoechst (dilution 1:1000). The cells were imaged using an Olympus IX73 microscope (**A**). Additionally, using the CellSens software, the mean gray value was determined for 30 representative cells (**B**). A representative result of three independent experiments is presented. In order to assess the significance between the samples, an ANOVA with Dunnett’s multiple comparisons post-hoc test was used. The results were considered statistically significant at *p* < 0.05 (* *p* ≤ 0.0332, *** *p* ≤ 0.0002, **** *p* < 0.0001).

The other question is a possible benefit for fungal cells from such surface HNE binding during host cell colonization. It is known that the destruction of host tissues promotes their settlement by pathogens [45]; however, the role of NET proteins bound to *C. albicans* cells and used against host epithelia was never considered. In order to test that, the purified HNE (14 µg per well) or the whole proteinous extract from NETs (212 µg per well) after DNA removal, were allowed to make contact with fungal hyphae and yeast cells before settling them on the monolayer of the A549 epithelial cell line. The used protein amounts corresponded to those detected during experiments on yeast cells’ contact with whole components of netting neutrophils. After 24 h of host cell treatment with (i) purified NET proteins or (ii) camptothecin as a positive control, and (iii) fungal cells modified by adhesion of NET-originated proteins, the changes in the viability of the host cells were studied using a MitoTracker stain (Figure 7). The quantitative analysis of images (Figure 7A) interpreted as a change in epithelial cell viability (Figure 7B) due to the contact with a solution of pure HNE or NET proteins or their *C. albicans* cell-surface-bound form, indicated that their adsorption on the fungal surface allowed for a significant increase in the intensity of host local cell damages compared with the action of host protein-free *C. albicans* cells. Such an application of neutrophilic proteins by invading *C. albicans* cells may unexpectedly promote yeast infections. 

### 3.5. Is the Modification of NET-Composing Proteins Responsible for C. albicans Cell Release from NET Structures? 

Still, an intriguing issue seems to be the fate of histones of which the binding to yeast cells was identified in the studies using commercial proteins or proteins produced in response to PMA, but their presence was not detected on the surface of *C. albicans* cells contacting netting host cells. In order to address this problem, we considered possible modifications of host proteins by enzymatic processes occurring at the place of the neutrophils’ responses to *C. albicans* invasion, which can influence the mutual interactions. 

Looking from the side of the host responses, the important modification of proteins occurring at the place of NET release is their citrullination, resulting from Pad4 activation [46,47]. However, the occurrence of Pad4-mediated modification depends on the type of stimulant and netosis progress. Hence, we can come across a variable proportion of both citrullinated and uncitrullinated proteins. Using PMA as the NET trigger, we can expect primarily uncitrullinated NET-proteins, but in response to *C. albicans,* the proteins were shown to be mostly modified [48]. Such modification was presented for histones and peptide LL-37 during NET analysis [47,49,50]. However, the studies were inconclusive in regard to Pad4 involvement in this process during neutrophil contact with *C. albicans* [51]. Independent of the rate of histone citrullination during neutrophil contact with *C. albicans* cells, both forms of these proteins can be bound to their surface, without any changes in the binding properties, as was demonstrated for the strongest histone H3 binding partner, Als3 (Supplementary Figure S4). 

**Figure 8:**
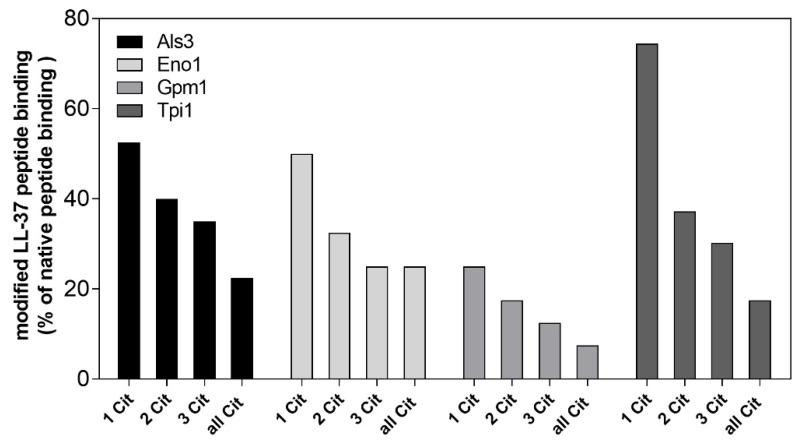
Influence of LL-37 citrullination on its binding properties towards the fungal adhesin Als3 and moonlighting proteins: Eno1, Tpi1, and Gpm1. The fungal proteins immobilized on the CM5 chips were tested for peptide LL-37 binding using the BIACORE 3000 system and SPR signal analysis (see Figure 5). The data from three independent tests were analyzed by BIAevaluation software, version 4.1 (GE Healthcare). The binding properties were compared between native LL-37 peptide (100%) and commercially available peptides with arginine residues converted to citrulline, at the places where citrullination was identified and described to be presented during netosis: 1 Cit, LL-37(7)Cit; 2 Cit, LL-37(7,29)Cit; 3 Cit, LL-37(7,29,34)Cit; and all Cit, LL-37(7,19,23,29,34)Cit.

We also analyzed the issue of citrullination using LL-37 and its modified structure to determine the role of arginine modification in its binding properties towards fungal partners, wherein its cationic charge and the structure of the peptide is important, but can be abrogated upon citrullination (Figure 8). The analysis was performed using the most sensitive SPR methods and fungal proteins immobilized on the chip surface. The gradual modification of arginine residues of the LL-37 peptide which were identified as the target for Pad4 [52,53] resulted in the loss of its ability to interact with all of the identified fungal proteins.

The most sensitive protein to LL-37 citrullination seemed to be Gpm1, as the modification of only one arginine residue in the LL-37 molecule led to the loss of about 75% of its binding affinity to Gpm1, but this was quite expected due to the weakest bounding properties of Gpm1 towards LL-37 among all of the tested proteins. On the other hand, the interaction of modified LL-37 with its strongest binder, Eno1, as well as the fungal adhesin Als3 resulted in a 50% decrease of its binding capacity after R7 modification. In the case of Tpi1, a gradual loss of its ability to interact with LL-37 was observed in correlation to the increase of the citrulline residue number. As the discussed modification changed the positive charge of each arginine residue to neutral citrulline, the resulting decrease in fungal surface protein binding pointed to the primarily electrostatic interaction of LL-37 with the selected proteins.

**Figure 9:**
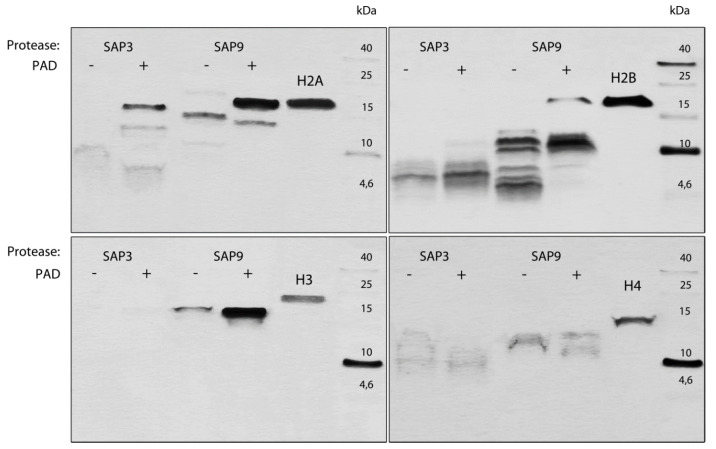
The influence of histone citrullination on the progress of histone degradation by *C. albicans* aspartic proteases. Unmodified (−) and deiminated histones (+) were treated with selected, recombinant aspartic proteinases (Saps, 6 nM) at a 1:1000 enzyme:substrate molar ratio (pH 5, 37 °C, 1 h). The reaction was stopped with pepstatin A (10 µM). The degradation profile was analyzed using SDS-PAGE in the Schägger von Jagov system with silver staining after glutaraldehyde fixation for the protein band visualization, and the representative result of three experiments is presented.

Looking from the fungal perspective, the main reaction-modifying NET proteins and their interactions can involve secreted *C. albicans* enzymes, mainly aspartic proteases, which can lead to a degradation of NET proteins, causing the relaxation of neutrophil trapping forces. The upregulation of genes encoding these proteases during NET secretion on contact with *C. albicans* was shown in our earlier report, in which we also presented the possible proteolytic inactivation of LL-37 by Saps [54]. In order to verify this hypothesis, the proteins composing NETs were treated with recombinant Sap enzymes at pH conditions simulating the different types of physiological niches where NETs can occur (Figure 9). The degradation profile of NET proteins pointed out histones next to LL-37 as the most potent components of NETs, which are sensitive to Sap degradation. In the same conditions, MPO, HNE, and LF were resistant to proteolysis. Among the tested proteases, Sap 3 and Sap 9 were selected as the most effective, and are still presented at the infection place. However, their operating efficiency can differ depending on the infection niche and its local pH. Among the analyzed histones, histones H3 and H4 seemed to be more prone to degradation, but their possible citrullination by Pad4 partially protected them.

In summary, both types of described modifications of NET-forming proteins―citrullination and the proteolytic degradation of histones and peptide LL-37―can modulate the effectiveness of NET-trapping and -killing properties, which in turn can influence the *C. albicans* cells’ survival.

## 4. Discussion

The critical role in the control and limitation of infection caused by *C. albicans* was attributed to neutrophils, as evidenced, e.g., from the observation of increased mortality rates and failure to clear fungal infection in patients with neutropenia [55]. The mutual contact of *C. albicans* cells and polymorphonuclear leukocytes (PMNs) is a dynamic process under which the recognition of fungal surface components prompts neutrophils, via the action of proper surface receptors, to various responses [56]. PMNs can apply the intra- and extracellular antimicrobial activities [57] using mainly the phagocytosis mechanism towards a part of the fungal cells, or arresting them mostly in the yeast form [55]. The remaining living microbial cells after early contact with neutrophils may alter the availability of cell wall epitopes to affect subsequent immune responses [21]. The filamentous fungal form, which is more resistant to host defense and masking its inflammation-evoking epitopes from host recognition [58], is partially killed by excreted NETs, with calprotectin as the main antifungal component of this phenomenon [38]. However, the efficiency of neutrophil fungal-killing properties seems to depend not only on the efficient functioning of immune cells but also on the dynamic mobilization of fungal cell wall machinery in response to PMN attack [59]. The plasticity of the fungal cell wall, identified during the surface proteome analysis of *C. albicans* upon contact with different types of host cells, has shown a changing response to host immune cells during mutual interactions [60,61]. 

Fungal hyphae, on the initial contact with neutrophils, can adopt the cell surface exposition of more resistant chitin [62] or overexpress some rescuing proteins, such as Sod5, to fight the generated oxidative stress [55]. The observed fungal cell wall remodeling on contact with NETs also concerned the promoted exposition of β-glucans, which can enhance the immune recognition of the invader by dedicated receptors [59]. However, nothing is known about the role of the surface proteins of *C. albicans* cells trapped within the NET structure, or about the possible evolution of such interactions during further host–microbe coexistence.

As was proposed, the antimicrobial function of NETs is provided by the trapping and localization of pathogens within a sticky mass of de-condensed chromatin as well as by the exposure of invaders to locally concentrated antimicrobial factors [63]. However, the exact mechanism of this phenomenon is still unknown. It was commonly accepted that the DNA phosphodiester backbone of NETs could be responsible for microbe trapping or killing. The latter could be a consequence of metal cation sequestration that resulted in bacterial membrane destabilization [64]. In contrast, the low concentration of neutrophil DNA detected by the bacterium *P. aeruginosa* leads to the upregulation of the bacterial genes required to modify the bacterial outer membrane surface and, thus, to increase the microbial tolerance for the toxic effects of NETs [65].

In order to examine whether the decondensed chromatin or granular proteins that compose NETs are responsible for trapping the fungal cells, we used the models in which NETs were treated with DNase or proteases, and further tested for *C. albicans* cell binding. The results pointed rather to the proteinous components of NETs as crucial molecules in this process. A surprisingly low impact of DNA on the immobilization of the fungal cells presented in the experiments on DNA degradation can reflect the weaker interactions between DNA and fungal cells compared with the interaction with NET’s proteinous components. It is also possible that NETs released directly at the place of infection in the response to fungal cells could also add to the NET binding properties the mechanical trapping or entwining of fungus by the released DNA web. 

In order to identify the possible interactions between the main proteinous components of NETs and the fungal cell’s surface, or directly, the interactions with selected candidal surface proteins, different approaches were used. First, the microscopic and MS analysis of NET-composing proteins that stay located or bound to the *C. albicans* cell surface indicated MPO, LF, HNE, cathepsin G, histones, calprotectin, and cathelicidin fragments (peptide LL-37) as potent partners in the interactions with fungal surface proteins. In order to verify this assessment, the binding of fluorescently-labeled pure neutrophil proteins, as selected above, was tested in contact with the whole *C. albicans* cell in both morphological forms, confirming the importance of the fungal cell sequestration of histones and the main granular proteins, with these being previously involved in the initial steps of NET formation [66], with the strongest binding properties for MPO to both morphological cell types of *C. albicans*. 

In order to clarify this problem, we performed the analysis of *C. albicans* surface-exposed proteins that changed during the contact of *C. albicans* cells with netting neutrophils [56]. The proteomic analysis pointed to changes within the main fungal adhesin belonging to the Als-family and involved in the binding of several host proteins [33], as well as in the group of “moonlighting” proteins commonly represented in the fungal biofilms by Eno1, Tpi1 and Gpm1 [67,68,69], which, besides their intercellular functions, can also operate on the fungal surface, interacting with host epithelial and phagocytic cells, as well as with mucins and circulating proteins of the immune and hemostatic systems [28,33,43,70]. The contribution of moonlighting proteins to the interaction with NET proteins was confirmed by crosslinking methods using NET proteins as a bait. However, such an approach did not detect any typical surface adhesins interacting with the proteinous NET component. Having in mind the high glycosylation of Als3 molecules and the possible involvement of the non-proteinous part of Als in the interaction with NET proteins, such results could not definitely exclude the Als roles in this process. In order to overcome this problem, we used *S. cerevisiae* cells exposing Als3 molecules on the yeast cell surface, and found Als3 to be the main target for the interaction with MPO and histone H3, which was also confirmed by the determination of the binding parameters between these purified proteins. The Als3 involvement is not surprising, as Als family proteins play a major role in *C. albicans’* adhesive properties [71,72], with the largest contribution in the adhesion to human cells [73], engaging the N-terminal part of its molecule. Moreover, Als, together with moonlighting proteins such as enolase, may participate in regulation of the binding of human proteins to the surface of yeast cells [33]. Thus, the problem of the role of the Als family in response to NETs remains for more detailed consideration.

The next group of fungal proteins involved in the binding of NET-composing proteins was constituted by moonlighting proteins, including *C. albicans* enolase, which belongs to the cytosolic proteins identified abundantly on the fungal cell surface [74], and for which a function of an immunodominant antigen detected during systemic fungal infections was also designed [75]. An increased exposition of enolase on the fungal cells contacting netting neutrophils was identified in our MS analysis and its gene expression increased during contact with netting neutrophils was also detected by Niemiec et al. [56]. In the approach presented here, enolase was identified as the strongest binder for antifungal LL-37 peptide and for HNE, a neutrophil serine protease, which retained the enzymatic activity upon binding. However, its proteolytic properties did not influence the mutual interactions. Enolase also belonged to the fungal proteins involved in the interaction with histone H4, but its binding characteristics indicated much weaker interactions compared with granular proteins. Enolase was also shown to bind several host proteins, including plasminogen [33,43,76] and the contact system proteins (high-molecular-mass kininogen, coagulation factor XII and plasma prekallikrein) [28,77,78]. Thus, its universal binding properties can also be adopted by *C. albicans* during contact with netting neutrophils. 

Gpm1 is another enzyme involved in the glycolytic pathway which moonlights at the candidal surface, shown here to bind another neutrophil granular protein, i.e., LF presented in the NETs. Gpm1 was also a binding partner for histone 3, which presented the strongest binding to *C. albicans* cells among all of the analyzed histones. Gpm1’s binding properties were more modest compared with enolase, and it was identified as a binding partner for human keratinocytes and human umbilical vein endothelial cells interacting with vitronectin and fibronectin [79]. Gpm1 was also identified to be involved in the binding of plasminogen [80] and proteins composing the contact system [28]. The next identified fungal moonlighting protein of *C. albicans*, also a glycolytic enzyme, was Tpi1, which was identified as a receptor for two proteins of the kinin-generated system: kininogen and factor XII [28]. Its binding properties on different *Candida* species were also documented towards human extracellular matrix proteins [81]. Tpi1 can also be involved in the interaction with NET proteins—mainly MPO, lactotransferrin and cathelicidin LL-37—but with a lower affinity than the above-named fungal binders. 

**Figure 10:**
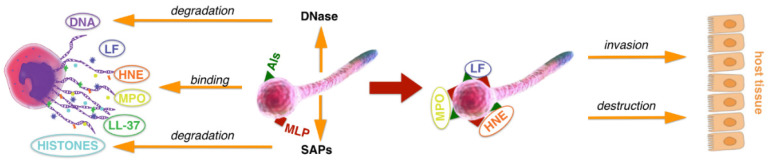
Proposed mechanisms of the possible usage of *C. albicans* adhesins and secreted enzymes in the invasion and defense of fungal cells against netosis. Secreted enzymes: DNase, endonucleases used in neutrophil DNA degradation; SAPs – secreted aspartic proteases degrading host proteins and antimicrobial peptides. Surface proteins: Als, adhesins of a family of agglutinin-like sequence proteins; MLP, moonlighting proteins, cytosolic enzymes with additional surface adhesive properties, including among others Eno1 (enolase), Gpm1 (phosphoglycerate mutase), Tpi1 (triosephosphate isomerase). Host proteins identified to be bound to *C. albicans* cells during NET release by neutrophils: HNE, neutrophil elastase; MPO, myeloperoxidase; LF, lactotransferrin; LL-37, an antimicrobial peptide derived from cathelicidin; HISTONES: H2, H3, H4.

However, the binding of homicidal factors to the surface of yeast cells during netosis seems to be a suicidal activity for them. This contradicts the information that fungal cells possess the ability to escape from the trapping by NETs, a feature attributed to the action of extracellular endonucleases presented in different yeast strains, which could be involved in the slow release of still-viable or better-adopted fungal cells, which are capable of spreading further and infecting the host organism [82]. Therefore, we performed the analysis of a fate of proteins associated with the *Candida* cell surface and their possible effects on host cells in order to present a possible scenario in which these host proteins can be eliminated or inactivated, or even used in further infection (Figure 10). 

Our analysis pointed out the histones as the potential ligands for *C. albicans* CWP, although with a low binding affinity, in which Als3 and moonlighting proteins Gpm1 for histone H3 and Eno1 for histone H4 were involved. Histones, representing ca. 70% of the total proteinous component of NETs, come from chromatin decondensation [38]. Upon contact with *C. albicans* cells, the citrullination of histone H3 was identified, as a result of the action of the deiminase Pad4, which is responsible for the conversion of arginine residue into citrulline [83]. The comparison of the binding properties between native and citrullinated histone H3 performed in this study did not show any significant citrullination influence on the histone interaction with fungal proteins. Histones are assigned the main antibacterial activity [84], especially after proteolytic processing by elastase during nucleus decondensation, as discussed by Papayannopoulos et al. [18]. In our approach, we also considered the possible proteolysis of histones in their native or citrullinated form by *C. albicans* proteases (Saps) overexpressed during contact with NET-forming neutrophils [9]. Although the citrullination of histones lowered the rate of the proteolysis by Saps, the resulting peptides probably did not significantly influence the *C. albicans* viability, as their antifungal properties in NETs were questioned by Urban et al. [38,85]. Whether it is a consequence of the arginine modification or degradation with the action of *C. albicans* Saps or neutrophil granular proteinases remains to be clarified.

An opposite consequence of the citrullination process on the binding to fungal surface proteins was identified for an amphipathic, positively-charged peptide, LL-37, generated from the cathelicidin hCAP18 and identified as a NET component [11]. Its effectiveness towards *C. albicans* cell killing [86] was attenuated by fungal proteolytic Sap-dependent activity, but its inflammatory regulation properties, like neutrophil chemotaxis, ROS generation, IL-8 release and the inhibition of neutrophil apoptosis [54], were fully eliminated from the hereby analyzed phenomena. On the other hand, the progressive citrullination of the arginine residues of LL-37 endured all of the proteinous interaction with fungal cell wall exposed proteins, similarly to that found in the case of LL-37-dependent nucleic acid recognition by host cells, where the interaction of DNA with citrullinated LL-37 resulted in the diminished activation of dendritic cells and macrophages [49]. This type of LL-37 modification also lowered the LL-37-mediated neutralization of lipopolysaccharide’s pro-inflammatory activity, decreasing its affinity for endotoxin [53].

The next neutrophil protein identified in our work as being important for interaction with *C. albicans* is LF, the well-known antimicrobial protein, the antifungal activity of which was attributed to iron sequestration, resulting in a fungistatic effect [87]. However, some other studies presented the increase in *Candida* species’ cell surface permeability after a direct interaction of LF with the fungal cell surface, leading to cell death [88,89]. 

LF detected as a NET component originates from the neutrophil secondary granules, but its significance for NET formation remains unclear [38]. The binding of LF to the selected fungal proteins offers the possibility of its lethal action both against its yeast partner and against neighboring host cells, as demonstrated in this work for the global effect of *C. albicans* alone versus NET-protein bound *C. albicans*. 

The most interesting NET proteins bared by *C. albicans* cells are HNE and MPO, the enzymes influencing chromatin decondensation [90,91]. As we determined, the *C. albicans* surface proteins possess the strongest binding properties towards MPO with the lowest K_d_ (11.6 nM) characterizing its interaction with the main fungal adhesion, Als3. Four-fold weaker interactions of MPO were noted for non-covalently bound fungal surface proteins like Eno1 and Tpi1. This is the first study that points to fungal proteins as MPO interaction partners. Previous experiments performed by Wright et al. [92] indicated mannans in plasma and tissue fluid as the binding ligands for MPO in *C. albicans*-infected patients, but using its soluble form they identified inhibitory effects towards MPO activity. On the other hand, the interactions between MPO and the identified proteinous fungal partners did not influence the MPO’s enzymatic properties, meaning that at the place of infection MPO could still be responsible for the conversion of hydrogen peroxide to hypochlorite used for microbe inactivation [93,94]. Although the selective binding of MPO to a microbe guarantees the proximity of the enzyme to the target [95], the reaction’s efficiency requires an acidic environment. Such acidification at areas of inflammation was assigned to massive infiltration by immune cells [96,97], and was identified at sites of autoimmune inflammation, such as the synovial fluid from joints of patients with rheumatoid arthritis [98]. On the other hand, Behnen et al. [99] showed that an acidic inflammatory environment results in the inhibition of extracellular neutrophil mechanisms such as the release of ROS and NETs. Moreover, *C. albicans* possesses an effective mechanism of defense, with ROS using superoxide dismutase Sod 5 localized to the fungal cell surface, in cooperation with catalase. However, both genes encoding these defense enzymes showed lower expression levels during one hour of contact with neutrophils resulting in NET formation [56]. 

On the other hand, the exposure of numerous types of host cells to the damaging-in-nature MPO products causing the cell death occurred via different pathways (see reviews [100,101]), influencing the cell membrane with final cell lysis, as demonstrated for red blood cells [102], or damaging the mitochondria of monocytes and macrophages with their necrotic death [103]. Furthermore, endothelial cells’ contact with MPO activity resulted in mitochondrial dysfunction and necrosis or apoptotic cell death [104]. A similar cytotoxicity of MPO was also reported for lung and bronchial epithelial cells [105], fibroblasts [106], and vascular smooth muscle cells [107]. Thus, it can be assumed that the MPO bound to the fungal cell surface may serve the pathogens to cause damage to the surrounding host tissue, which may be beneficial for the further infection propagation, especially when the fungal protection mechanisms are secured at the same time. Such a sophisticated mechanism was observed in the case of contact between some Gram-negative and Gram-positive bacteria, where the low MPO binding streptococci, being the source of H_2_O_2,_ used the synergistic action of MPO strongly bound to the cells of *E. coli*, *S. aureus*, or *P. aeruginosa*, to eliminate the competitors at the infection place [108]. 

Another important NET-composing protein bound to the surface-exposed fungal proteins is HNE, which can directly kill bacterial cells by the cleavage of its outer membrane components [109,110], or by attenuating bacterial virulence factors like bacterial flagellin [111] or leukotoxin [112,113]. The activity of HNE seems to be preserved by the enzyme bound to DNA fiber formed during netosis, as was shown by Kolaczkowska et al. [57], but its specific activities may depend on its exact location and possible electrostatic interactions [114]. Although a bacterium like *S. aureus* produces inhibitory HNE-binding proteins [115], its activity was still maintained during the elastase binding to selected fungal proteins—Als3 and the analyzed moonlighting proteins—and, as we presented in this work, resulted in the fungal cells’ mortality rate of 25% after 24 h of contact. On the other hand, we identified that binding between HNE and Als3 is sensitive to proteolysis when Als3 was overexpressed in *S. cerevisiae*. The proteolytic processing was not observed using the Als3 isolated from the surface of *C. albicans*. This could suggest that the proper post-translation modification of Als3 can equip it with a resistance to degradation by host proteases. Such a strategy was identified in *S. epidermidis* and *S. aureus* cells which express glycosyltransferases, decorating cell-surface-bound proteins with N-acetylglucosamine moieties, which protect them from proteolytic degradation [116].

On the other hand, HNE associated with *C. albicans* surface proteins can lead to the increased destruction of human cells in comparison to the infection caused by this pathogen itself. Thus, we indicated for the first time that *C. albicans* cells, after breaking free from NETs through DNA degradation, may use the released neutrophil proteins produced during netosis to increase the virulence of their cells in the process of host tissue colonization by the still-alive part of the pathogen population.

In summary, this report highlights that the NET-trapped fungal pathogen, *C. albicans*, exposes on the cell surface the proteins belonging to typical adhesins and to the group of moonlighting proteins, which together are involved in the interactions with host proteins submerged in NETs’ structures. The possible elimination of NET-forming DNA structures by fungal DNases produced by different *Candida* strains [82] does not influence the strong interaction of fungal cells with NET proteins, preserving their destructive activity, which can be directed against the surrounding host tissues. Overall, these insights will contribute to a better understanding of the roles of fungal cell surface changes and the role of the surface deposition of the cytosolic proteins in the fungal survival strategies. Moreover, the results indicate the need to look for new strategies which might offer the greatest efficacy in preventing fungal-associated tissue damage concerning the shedding of fungal, cytosolic molecules anchoring host proteins and possibly used in infection propagation.

## Figures and Tables

**Table 1:** Comparison of the binding parameters determined for the complexes formed between isolated *C. albicans* surface-exposed proteins and selected host proteins composing NET structures. The K_d_ values were determined using the SPR technique applied in the BIACORE 3000 system, in which fungal proteins were immobilized on the surface of CM5 chips, reflecting the surface-immobilized nature of these proteins. The solutions of host proteins at the wide concentration range were allowed to make contact with the chip-immobilized fungal proteins. The association and dissociation processes were identified as was described in the legend to Figure 5. The data from three independent tests were analyzed by BIAevaluation software version 4.1 (GE Healthcare). For each dataset, the simultaneous fitting of the association and dissociation rate constants and the equilibrium constant was used with the 1:1 Langmuir binding model. (*) The binding parameters for all of the histones were determined by the ELISA-like method, in which the microplate-immobilized fungal protein was allowed to bind soluble fluorescent-labeled host proteins. The binding analysis was performed using GraphPad Prism software version 8.0 and a saturation one-site binding model (nd, not determined; nb, not bound).

NET Proteins	Cell Wall Proteins of *C. albicans* (K_d_ (nM))			
	Als3	Eno1	Gpm1	Tpi1
**MPO**	11.6 ± 6	78.7 ± 45	353 ± 13.2	45 ± 0.2
**HNE**	65 ± 5.3	36.1 ± 0.5	nd	nd
**LF**	85.9 ± 1.9	72.2 ± 0.1	21.4 ± 0.2	163 ± 0.2
**LL-37**	228 ± 54	14.6 ± 0.5	598 ± 2.8	68.3 ± 0.1
H2 *	1787 ± 238	911 ± 238	nb	nd
H3 *	23.4 ± 8.6	1234 ± 534	258 ± 78	nd
H4 *	820 ± 330	384 ± 130	1898 ± 414	nd

## Data Availability

The data presented in this study are included in the article and Supplementary Materials. Further inquiries should be directed to the corresponding author.

## Supplementary Materials

The following are available online at https://www.mdpi.com/article/10.3390/cells10102736/s1: Figure S1. Comparison of interaction properties between *S. cerevisiae* strains exposing Als3 and its mutated form Als3Δ325-331towards selected NET- composing protein–lactotransferrin. Figure S2. The influence of Mg2+ on interaction properties of enolase and MPO–an example. Figure S3. The influence of formation of complex between selected fungal proteins and MPO or HNE on their enzymatic activities. Figure S4. The comparable binding properties between Als3 and native or citrullinated histone H3. Table S1. The list of the *Saccharomyces* cerevisiae strains used in this study. Table S2. Mass spectrometric identification of human proteins associated to the fungal cell surface after interactions of *C. albicans* cells with isolated NETs. Table S3. Mass spectrometry identification of *C. albicans* cell wall-associated proteins binding particular NET proteins. Table S4. Mass spectrometric identification of fungal cell wall-associated proteins present at the fungal cell surface during the interactions of *C. albicans* with neutrophils.
