# Supplementary material for: Proteinous Components of Neutrophil Extracellular Traps Are Arrested by the Cell Wall Proteins of Candida albicans during Fungal Infection, and Can Be Used in the Host Invasion
Source: Cells. 2021 Oct 13;10(10):2736. doi: 10.3390/cells10102736 (PMC8534323; doi:10.3390/cells10102736)
Supplement: Supplementary file 1 [file cells-10-02736-s001.zip › cells-1405465-supplementary.pdf]

**SUPPLEMENTARY MATERIALS**

**Figures:**

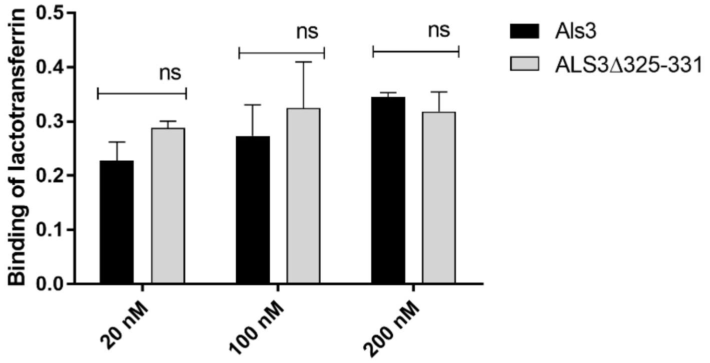


**Supplementary Figure S1.** Comparison of interaction properties between *S. cerevisiae* strains exposing Als3 and its mutated form Als3Δ325-331towards selected NET- composing protein – lactotransferrin.

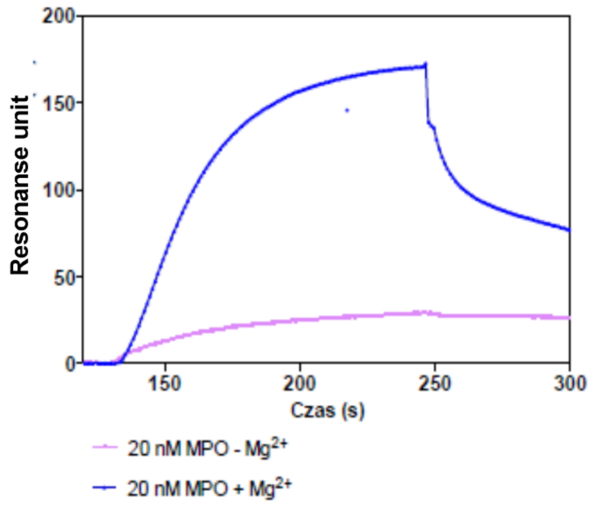


**Supplementary Figure S2.** The influence of Mg++ on interaction properties of enolase and MPO – an example.

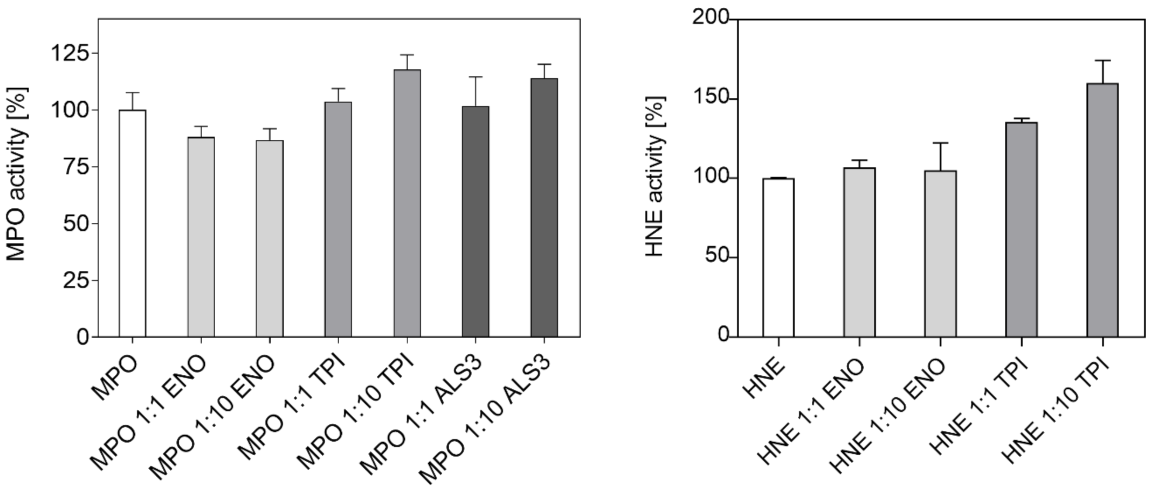


**Supplementary Figure S3.** The influence of formation of complex between selected fungal proteins and MPO or HNE on their enzymatic activities.

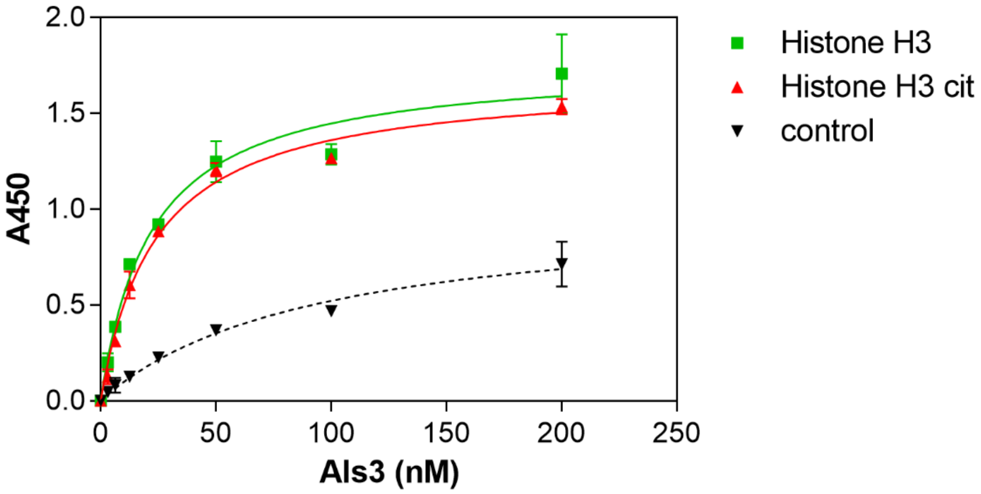


| | Histone H3 | Histone H3 cit | control |
|---|---|---|---|
| One site binding (hyperbola) | | | |
| Best-fit values | | | |
| Bmax | 1.764 | 1.682 | 1.008 |
| Kd | 21.97 | 23.78 | 93.36 |
| Std. Error | | | |
| Bmax | 0.08213 | 0.05074 | 0.0955 |
| Kd | 3.323 | 2.282 | 18.76 |

**Supplementary Figure S4.** The comparable binding properties between Als3 and native or citrullinated histone H3.

## Tables:

**Supplementary Table S1. The list of the *Saccharomyces cerevisiae* strains used in this study.**

| Strain | Description | Reference |
|---|---|---|
| BY4742<br>UB2022 | *MATα leu2Δ0 lys2Δ0 ura3Δ0 his3Δ1* | (Brachmann et al., 1998) |
| UB2155 | BY4742 with pBC542<br>(9.2 kb; ApR; pMB1 *ori*; *URA3*) | (Nobbs et al., 2010) |
| UB2157 | BY4742 with pBC542-*ALS3lg* | (Nobbs et al., 2010) |
| UB2161 | BY4742 with pBC542-*CWP1* | (Nobbs et al., 2010) |
| UB2316 | BY4742 with pBC542-*ALS3Δ325-331* | (Bamford et al., 2015) |

Brachmann CB, Davies A, Cost GJ, et al (1998) Designer deletion strains derived from *Saccharomyces cerevisiae* S288C: a useful set of strains and plasmids for PCR-mediated gene disruption and other applications. Yeast Chichester Engl 14:115–132. https://doi.org/10.1002/(SICI)1097-0061(19980130).
Nobbs AH, Margaret Vickerman M, Jenkinson HF (2010) Heterologous expression of *Candida albicans* cell wall-associated adhesins in *Saccharomyces cerevisiae* reveals differential specificities in adherence and biofilm formation and in binding oral *Streptococcus gordonii*. Eukaryot Cell 9:1622–1634. https://doi.org/10.1128/EC.00103-10.Bamford CV, Nobbs AH, Barbour ME, et al (2015) Functional regions of *Candida albicans* hyphal cell wall protein Als3 that determine interaction with the oral bacterium *Streptococcus gordonii*. Microbiol Read Engl 161:18–29. https://doi.org/10.1099/mic.0.083378-0.

**Supplementary Table S2. Mass spectrometric identification of human proteins associated to the fungal cell surface after interactions of *C. albicans* cells with isolated NETs.**

To obtain the yeast-like form of *C. albicans*, cells were initially cultured at 30°C for 16 h in YPD medium, and to achieve filamentation 5x10$^{8}$ cells were transferred to RPMI 1640 medium and further incubated at 37°C for 3 h. Then 5x10$^{8}$ blastospores or cells in filamentous form wereincubatedat 37°Cfor 1 hinPBSbuffer with NETs isolated from 8x10$^{6}$ neutrophils. After washing out of unbound proteins, cell surface shaving with trypsin was performed as described previously in Karkowska-Kuleta et al. 2019 andobtained peptideswere analyzed by LC-MS/MS with HCT Ultra ETD II mass spectrometer (Bruker, Bremen, Germany) or Q-Exactive mass spectrometer (Thermo Scientific, Waltham, MA) – the parameters obtained from the latter are in italics. The obtained lists of peaks were searched against the SwissProt protein database (SC – sequence coverage).

| Accession Number | Protein Name | Score | Matches | SC [%] |
|---|---|---|---|---|
| **Yeast-like Cells** | | | | |
| ACTB_HUMAN | Actin, cytoplasmic 1 | 252 | 15 | 37 |
| | | 216 | 13 | 29 |
| | | *430.50* | *9* | *26.40* |
| ACTN1_HUMAN | Alpha-actinin-1 | 46 | 1 | 1 |
| | | 55 | 1 | 1 |
| | | *126.90* | *4* | *4.82* |
| BPI_HUMAN | Bactericidal permeability-increasing protein | *199.66* | *2* | *4.31* |
| C5AR1_HUMAN | C5a anaphylatoxin chemotactic receptor 1 | *35.40* | *1* | *2.86* |
| CAMP_HUMAN | Cathelicidin antimicrobial peptide | *90.85* | *3* | *13.53* |

| | | | | |
|---|---|---|---|---|
| CATG_HUMAN | Cathepsin G | 81 | 4 | 12 |
| | | 98 | 4 | 12 |
| | | *323.00* | *5* | *19.61* |
| DEF1_HUMAN | Neutrophil defensin 1 | *98.71* | *2* | *19.15* |
| ELNE_HUMAN | Neutrophil elastase | 57 | 2 | 6 |
| | | *96.48* | *2* | *6.37* |
| GELS_HUMAN | Gelsolin | 55 | 1 | 1 |
| | | *67.67* | *2* | *3.96* |
| H2AV_HUMAN | Histone H2A.V | 89 | 3 | 20 |
| | | 62 | 3 | 20 |
| H2AZ_HUMAN | Histone H2A.Z | *70.87* | *2* | *12.50* |
| H2B1B_HUMAN | Histone H2B type 1-B | 192 | 74 | 25 |
| H2B1K_HUMAN | Histone H2B type 1-K | *472.69* | *5* | *40.48* |
| H2B1C_HUMAN | Histone H2B type 1-C/E/F/G/I | 205 | 94 | 40 |
| H31_HUMAN | Histone H3.1 | *78.31* | *2* | *10.29* |
| H31T_HUMAN | Histone H3.1t | 51 | 5 | 23 |
| H33_HUMAN | Histone H3.3 | 41 | 6 | 24 |
| H4_HUMAN | Histone H4 | 90 | 5 | 49 |
| | | 103 | 4 | 29 |
| | | *225.11* | *4* | *40.78* |
| HBA_HUMAN | Hemoglobin subunit alpha | 38 | 1 | 6 |
| | | 38 | 1 | 6 |
| | | *97.80* | *1* | *10.56* |
| HBB_HUMAN | Hemoglobin subunit beta | 68 | 3 | 19 |
| | | 120 | 4 | 29 |
| | | *231.34* | *5* | *35.37* |
| KLH35_HUMAN | Kelch-like protein 35 | 35 | 2 | 1 |
| LYSC_HUMAN | Lysozyme C | *74.36* | *1* | *8.11* |
| MNDA_HUMAN | Myeloid cell nuclear differentiation antigen | *105.30* | *2* | *5.90* |
| MRCKA_HUMAN | Serine/threonine-protein kinase MRCK alpha | 44 | 1 | 0 |
| MYH11_HUMAN | Myosin-11 | 48 | 2 | 0 |
| MYH9_HUMAN | Myosin-9 | 47 | 4 | 3 |
| | | *91.96* | *3* | *2.24* |
| PERM_HUMAN | Myeloperoxidase | 35 | 1 | 1 |
| | | *361.88* | *6* | *13.42* |
| PLSL_HUMAN | Plastin-2 | *271.11* | *8* | *19.46* |
| PRTN3_HUMAN | Myeloblastin | 96 | 4 | 17 |
| | | 40 | 3 | 7 |
| | | *214.97* | *4* | *20.70* |
| RAP1A_HUMAN | Ras-related protein Rap-1A | 79 | 3 | 12 |
| RS27A_HUMAN | Ubiquitin-40S ribosomal protein S27a | 41 | 1 | 10 |
| | | 52 | 1 | 10 |

| | | | | |
|---|---|---|---|---|
| S10A6_HUMAN | Protein S100-A6 | *34.86* | *1* | *8.89* |
| S10A8_HUMAN | Protein S100-A8 | 60 | 5 | 33 |
| | | 64 | 4 | 26 |
| | | *188.54* | *4* | *32.26* |
| S10A9_HUMAN | Protein S100-A9 | 127 | 6 | 37 |
| | | 113 | 6 | 50 |
| | | *474.74* | *6* | *56.14* |
| S10AC_HUMAN | Protein S100-A12 | *95.71* | *3* | *38.04* |
| TRFL_HUMAN | Lactotransferrin | *68.13* | *2* | *2.68* |
| VIME_HUMAN | Vimentin | 104 | 6 | 16 |
| | | 152 | 6 | 12 |
| | | *184.43* | *5* | *12.23* |
| **Hyphae** | | | | |
| ACTB_HUMAN | Actin, cytoplasmic 1 | 191 | 11 | 28 |
| | | 312 | 13 | 29 |
| | | *2927,79* | *7* | *56.80* |
| ACTN1_HUMAN | Alpha-actinin-1 | 132 | 4 | 4 |
| | | 72 | 2 | 3 |
| | | *2057.64* | *21* | *41.14* |
| BPI_HUMAN | Bactericidal permeability-increasing protein | *276.32* | *4* | *12.53* |
| C5AR1_HUMAN | C5a anaphylatoxin chemotactic receptor 1 | 49 | 1 | 2 |
| CAMP_HUMAN | Cathelicidin antimicrobial peptide | 38 | 2 | 10 |
| | | *65.59* | *2* | *10* |
| CATA_HUMAN | Catalase | *716.49* | *14* | *38.71* |
| CATG_HUMAN | Cathepsin G | 122 | 5 | 12 |
| | | 93 | 4 | 16 |
| | | *96.12* | *2* | *8.24* |
| DEF1_HUMAN | Neutrophil defensin 1 | *119.15* | *3* | *52.13* |
| ELNE_HUMAN | Neutrophil elastase | 75 | 2 | 6 |
| | | 80 | 2 | 6 |
| | | *84.05* | *1* | *3.75* |
| GELS_HUMAN | Gelsolin | *1436.32* | *22* | *45.01* |
| H2A1A_HUMAN | Histone H2A type 1-A | 56 | 3 | 17 |
| H2AY_HUMAN | Core histone macro-H2A.1 | *427.83* | *6* | *25.81* |
| H2AJ_HUMAN | Histone H2A,J | 74 | 4 | 26 |
| H2AZ_HUMAN | Histone H2A.Z | *258.28* | *2* | *31.25* |
| H2B1B_HUMAN | Histone H2B type 1-B | 271 | 10 | 39 |
| | | 332 | 8 | 33 |
| H2B1J_HUMAN | Histone H2B type 1-J | *2578.11* | *7* | *51.59* |
| H33_HUMAN | Histone H3,3 | 47 | 5 | 19 |
| | | 51 | 7 | 24 |
| | | *121.08* | *3* | *14.07* |

| | | | | |
|---|---|---|---|---|
| H4_HUMAN | Histone H4 | 93 | 3 | 29 |
| | | 88 | 3 | 29 |
| | | *86.41* | *3* | *31.07* |
| HBA_HUMAN | Hemoglobin subunit alpha | *386.01* | *7* | *66.90* |
| HBB_HUMAN | Hemoglobin subunit beta | 113 | 3 | 21 |
| | | *612.40* | *8* | *60.54* |
| KLH35_HUMAN | Kelch-like protein 35 | 35 | 1 | 1 |
| | | 35 | 1 | 1 |
| LYSC_HUMAN | Lysozyme C | 65 | 3 | 16 |
| | | *65.66* | *1* | *8.11* |
| MNDA_HUMAN | Myeloid cell nuclear differentiation antigen | *285.63* | *8* | *28.75* |
| MYH9_HUMAN | Myosin-9 | 41 | 1 | 0 |
| | | *4415.28* | *41* | *36.38* |
| PERM_HUMAN | Myeloperoxidase | *50.96* | *1* | *1.34* |
| PLSL_HUMAN | Plastin-2 | *1947.91* | *29* | *58.69* |
| PROF1_HUMAN | Profilin-1 | 71 | 2 | 20 |
| | | *874.17* | *6* | *57.14* |
| PRTN3_HUMAN | Myeloblastin | 71 | 1 | 9 |
| | | 74 | 3 | 17 |
| | | *213.36* | *4* | *20.70* |
| RAP1B_HUMAN | Ras-related protein Rap-1b | 150 | 4 | 20 |
| | | 128 | 3 | 20 |
| RL40_HUMAN | Ubiquitin-60S ribosomal protein L40 | 52 | 2 | 19 |
| S10A4_HUMAN | Protein S100-A4 | *129.15* | *2* | *18.81* |
| S10A6_HUMAN | Protein S100-A6 | 42 | 1 | 8 |
| | | *86.13* | *3* | *24.44* |
| S10A8_HUMAN | Protein S100-A8 | 42 | 5 | 33 |
| | | 66 | 5 | 32 |
| | | *2792.07* | *15* | *97.85* |
| S10A9_HUMAN | Protein S100-A9 | 195 | 8 | 52 |
| | | 197 | 9 | 67 |
| | | *16150.64* | *10* | *86.84* |
| S10AB_HUMAN | Protein S100-A11 | *244.12* | *4* | *39.05* |
| S10AC_HUMAN | Protein S100-A12 | *454.72* | *5* | *72.83* |
| STOM_HUMAN | Erythrocyte band 7 integral membrane protein | 81 | 2 | 8 |
| TRFL_HUMAN | Lactotransferrin | *57.28* | *1* | *1.41* |
| VIME_HUMAN | Vimentin | 144 | 6 | 12 |
| | | 206 | 9 | 16 |
| | | *1322.36* | *28* | *61.37* |

Karkowska-Kuleta J, Satala D, Bochenska O, et al (2019) Moonlighting proteins are variably exposed at the cell surfaces of *Candida glabrata*, *Candida parapsilosis* and *Candida tropicalis* under certain growth conditions. BMC Microbiol 19:149. https://doi.org/10.1186/s12866-019-1524-5.

**Supplementary Table S3. Mass spectrometry identification of *C. albicans* cell wall-associated proteins binding particular NET proteins.**

Chemical cross-linking was performed using trifunctional reagent sulfo-SBED (sulfo-N-hydroxysuccinimidyl-2-(6-[biotinamido]-2-(p-azido benzamido)-hexanoamido) ethyl-1,3'-dithioproprionate)(Thermo Fisher Scientific Inc., Woltham, MA, USA). Samples containing 50 μg of each NET-composing proteins in 20 mM HEPES buffer (pH 7.8) were treated with the 50-fold molar excess of cross-linker in the dark at 4 °C for 2 h. The samples were dialyzed against PBS at 4°C overnight in the dark to remove the unbound cross-linker. The particular labeled NET protein was incubated with a mixture of fungal CWP (300 μg in 300 μl PBS) at 37°C for 1 h in the dark. Then samples were placed on ice and exposed to UV radiation (365 nm) for 15 min. Covalently linked pairs of biotinylated proteins were adsorbed on MagnaBind Streptavidin Beads (Thermo Fisher Scientific Inc.). After 1 h of incubation with intense stirring, beads were washed five times with 1 ml of PBS to remove unbound proteins, followed by 1 h incubation with 40 μl of 50 mM DTT at 60°C. Samples were again washed 5 times with 1 ml of PBS. The fungal proteins were removed from the beads by boiling in 30 μl 2.5% β-mercaptoethanol and 2% SDS for 30 min. The released proteins were separated by SDS-PAGE, and protein bands were visualized by staining with Coomassie Brilliant Blue R-250 and analyzed by mass spectrometry as described in details in Seweryn et al. 2015.

| NET Protein | *C. albicans* Protein | MW (kDa) | Score | Identified Peptides | SC (%) |
|---|---|---|---|---|---|
| MPO | Eft2 | 93.9 | 594 | 16 | 29 |
| | Eno1 | 47.2 | 510 | 7 | 27 |
| | Tpi1 | 26.9 | 386 | 8 | 56 |
| LF | Eft2 | 93.9 | 302 | 10 | 17 |
| | Eno1 | 47.2 | 237 | 5 | 15 |
| | Gpm1 | 27.4 | 212 | 11 | 54 |
| | Tpi1 | 26.9 | 113 | 4 | 23 |
| H3.1 | Eno1 | 47.2 | 444 | 9 | 24 |
| | Eft2 | 93.9 | 385 | 10 | 13 |
| | Gpm1 | 27.4 | 311 | 11 | 50 |
| | Tpi1 | 26.9 | 112 | 4 | 17 |
| H2A | Eno1 | 47.2 | 410 | 10 | 29 |
| | Tpi1 | 26.9 | 196 | 8 | 38 |
| | Gpm1 | 27.4 | 163 | 7 | 37 |
| H4 | Eno1 | 47.2 | 583 | 9 | 26 |
| | Tpi1 | 26.9 | 271 | 11 | 56 |
| | Gpm1 | 27.4 | 236 | 6 | 28 |
| | Eft2 | 93.9 | 104 | 2 | 3 |
| HNE | Eno1 | 47.2 | 544 | 10 | 29 |
| | Tpi1 | 26.9 | 220 | 9 | 46 |

MW – molecular weight; SC – sequence coverage

Seweryn K, Karkowska-Kuleta J, Wolak N, et al (2015) Kinetic and thermodynamic characterization of the interactions between the components of human plasma kinin-forming system and isolated and purified cell wall proteins of *Candida albicans*. Acta Biochim Pol 62:825–35. https://doi.org/10.18388/abp.2015_1142.

**Supplementary Table S4. Mass spectrometric identification of fungal cell wall-associated proteins present at the fungal cell surface during the interactions of *C. albicans* with neutrophils.**

*C. albicans* cells (4x10$^{9}$), after washing with PBS were mixed with 4x10$^{7}$ of human neutrophils suspended in 5 ml of fresh RPMI 1640 medium and co-incubated with gentle stirring for 4 h in a humidified 5% $CO_2$ atmosphere at 37˚C. Fungal cells incubated under the same conditions but without neutrophils served as a control. After incubation, human and fungal cells were collected by centrifugation at 2000 rpm for 3 min at 4˚C, re-suspended in 5 ml of PBS buffer with calcium and magnesium ions and the 750 µl of the solution of micrococcal nuclease (MNase) (Roche, Basel, Switzerland) was added at concentration of 1 U/ml for 15 min at 37°C. After removing of the supernatant, 5 ml of PBS has been added to cells with 1 mM phenylmethanesulfonyl fluoride (PMSF) (Sigma). The human cells have been broken by drawing them up to 20 times in a syringe (21 gauge) and their remnants were removed from *C. albicans* cells suspension by extensive washing with 1 ml of distilled water containing 1 mM PMSF and centrifugation at 3 000 rpm for 5 min. Washed fungal cells were frozen at -80°C and after that transferred to the new vials with 0.5 ml of distilled water with 1 mM PMSF and disrupted with glass beads (diameter 0.5 mm) (Sigma) in Precellys Evolution homogenizer (Bertin Instruments, Montigny-le-Bretonneux, France) with 20 sec pulse, 6 300 rpm, repeated four times. The obtained cell wall fraction was harvested by centrifugation at 5 000 rpm for 3 minutes, washed two times with 0.5 ml of ice-cold distilled water with 1 mM PMSF and then four times with 0.5 ml of each of the following ice-cold solutions containing 1 mM PMSF: 5% NaCl, 3% NaCl, 1% NaCl and finally again with 0.5 ml of cold distilled water in order to remove cytoplasmic contaminants from the cell walls preparation. *C. albicans* cell wall proteins were extracted by boiling the cell walls in 150 µl of the solution of 2% SDS and 2% 2-mercaptoethanol in water for 0.5 h. Then, obtained protein sample was extensively dialyzed against water at 4˚C. To qualitative assessment of proteins present at the fungal cell surface the SDS-PAGE electrophoresis was performed followed by staining of the proteins with Coomassie Brilliant Blue R-250 and excision of all noticeable protein bands. The proteins were then in-gel digested with trypsin and identified with LC-MS/MS (Seweryn et al. 2015). The obtained lists of peaks were searched against the SwissProt protein database restricted for Fungi. Proteins detected in particular experimental conditions were indicated as "+".

| Accession Number | Protein Name | *C. albicans* with Neutrophils | *C. albicans* |
|---|---|---|---|
| 1433_CANAL | 14-3-3 protein homolog Bmh1 | + | + |
| ACEA_CANAX | Isocitrate lyase Icl1 | + | + |
| ACON_CANAL | Aconitate hydratase, mitochondrial Aco1 | + | + |
| ACS1_CANAX | Acetyl-coenzyme A synthetase 1 Acs1 | + | + |
| ACS2_CANAL | Acetyl-coenzyme A synthetase 2 Acs2 | | + |
| ACT_CANAX | Actin Act1 | + | + |
| ADH1_CANAX | Alcohol dehydrogenase 1 Adh1 | + | + |
| ADH2_CANAL | Alcohol dehydrogenase 2 Adh2 | + | + |
| ALF_CANAL | Fructose-bisphosphate aldolase Fba1 | + | + |
| ALS1_CANAL | Agglutinin-like protein 1 Als1 | | + |
| ALS3_CANAL | Agglutinin-like protein 3 Als3 | + | + |
| BGL2_CANAL | Glucan 1,3-beta-glucosidase Bgl2 | + | + |

| | | | |
|---|---|---|---|
| CHI2_CANAL | Chitinase 2 Cht2 | + | + |
| EF1A1_CANAL | Elongation factor 1-alpha 1 Tef1 | + | + |
| EF2_CANAL | Elongation factor 2 Eft2 | + | + |
| ENO1_CANAL | Enolase 1 Eno1 | + | + |
| G3P_CANAW | Glyceraldehyde-3-phosphate dehydrogenase Tdh1 | + | + |
| GBLP_CANAL | Guanine nucleotide-binding protein subunit beta-like protein Asc1 | + | + |
| HSP60_CANAL | Heat shock protein 60, mitochondrial Hsp60 | + | + |
| HSP71_CANAL | Heat shock protein Ssa1 | + | + |
| HSP75_CANAW | Heat shock protein Ssb1 | + | + |
| HSP90_CANAL | Heat shock protein 90 homolog Hsp90 | + | + |
| IF4A_CANAL | ATP-dependent RNA helicase eIF4A Tif1 | | + |
| IPYR_CANAL | Inorganic pyrophosphatase Ipp1 | | + |
| KAD2_CANAL | Adenylate kinase Adk1 | + | + |
| MCR1_CANAL | NADH-cytochrome b5 reductase 2 Mcr1 | + | + |
| MDHC_CANAL | Malate dehydrogenase, cytoplasmic Mdh1 | + | + |
| MIC60_CANAL | MICOS complex subunit MIC60 Mic60 | + | |
| NCE2_CANAL | Non-classical export protein 102 Nce102 | | + |
| PCKA_CANAX | Phosphoenolpyruvate carboxykinase [ATP] Pck1 | + | + |
| PDC1_CANAL | Pyruvate decarboxylase Pdc11 | + | + |
| PGK_CANAL | Phosphoglycerate kinase Pgk1 | + | + |
| PHR1_CANAL | pH-responsive protein 1 Phr1 | + | |
| PMA1_CANAX | Plasma membrane ATPase 1 Pma1 | + | + |
| PMGY_CANAL | Phosphoglycerate mutase Gpm1 | + | + |
| PRA1_CANAL | pH-regulated antigen Pra1 | + | |
| QCR2_CANAL | Cytochrome b-c1 complex subunit 2, mitochondrial Qcr2 | | + |
| RL10A_CANAL | 60S ribosomal protein L10a Rpl10A | | + |
| RS3A_CANAL | 40S ribosomal protein S1 Rps1 | + | + |
| RS4_CANAX | 40S ribosomal protein S4 Rps4 | + | + |
| SODM_CANAX | Superoxide dismutase [Mn], mitochondrial Sod2 | + | + |
| SUR7_CANAL | Protein SUR7 Sur7 | + | + |
| TPIS_CANAL | Triosephosphate isomerase Tpi1 | + | + |
| TSA1_CANAL | Peroxiredoxin Tsa1 | + | + |
| VDAC_CANAL | Mitochondrial outer membrane protein porin Por1 | + | + |
| YWP1_CANAL | Yeast-form wall Protein 1 Ywp1 | + | + |

Seweryn K, Karkowska-Kuleta J, Wolak N, et al (2015) Kinetic and thermodynamic characterization of the interactions between the components of human plasma kinin-forming system and isolated and purified cell wall proteins of *Candida albicans*. Acta Biochim Pol 62:825–35. https://doi.org/10.18388/abp.2015_1142.
